# Supplementary material for: Integrative network modeling reveals mechanisms underlying T cell exhaustion
Source: Sci Rep. 2020 Feb 5;10:1915. doi: 10.1038/s41598-020-58600-8 (PMC7002445; doi:10.1038/s41598-020-58600-8)
Supplement: Supplementary file 1 — Supplementary Figures and Tables. [file 41598_2020_58600_MOESM1_ESM.docx]

## Integrative network modeling reveals mechanisms underlying T cell exhaustion

Hamid Bolouri^1,*^, Mary Young^2^, Joshua Beilke^3^, Rebecca Johnson^2^, Brian Fox^2^, Lu Huang^4^, Cristina Costa Santini^5^, Christopher Mark Hill^2^, Anne-Renee van der Vuurst de Vries^2^, Paul T. Shannon^6^, Andrew Dervan^2^, Pallavur Sivakumar^2^, Matthew Trotter^5^, Douglas Bassett^2^ & Alexander Ratushny^2,*^

^1^Division of Human Biology, Fred Hutchinson Cancer Research Center, Seattle, WA 98109, USA. ^2^Bristol-Myers Squibb, Summit, NJ, USA. ^3^Formerly Celgene Corporation, Seattle, WA, USA. ^4^Formerly Celgene Institute for Translational Research Europe (CITRE), Seville, Spain. ^5^Celgene Institute for Translational Research Europe (CITRE), a Bristol-Myers Squibb Company, Summit, NJ. ^6^Institute for Systems Biology, Seattle, WA 98109, USA.

*Correspondence should be addressed to:
H.B. (tel: +1 206 667 2748, email: HBolouri@fhcrc.org) or A.R. (tel: +1 206 709 6059, email: aratushny@celgene.com)

# **Supplementary Table S1. Large-scale TCE network specifications**

| **Source** | **Target** | **Reference** | **EdgeType** |
| --- | --- | --- | --- |
| AKT1 | BACH2 | 1 | Inhibits |
| AKT1 | FOXO1 | 2,3 | Inhibits |
| AKT1 | MTOR | 4 | Promotes |
| AKT1 | PPARGC1A | 5 | Inhibits |
| AKT1 | SLC2A1 | 6 | Promotes |
| AKT1 | SLC2A2 | 6 | Promotes |
| AKT1 | SLC2A3 | 6 | Promotes |
| AKT1 | SLC2A4 | 6 | Promotes |
| AKT1 | SLC2A5 | 6 | Promotes |
| AKT2 | BACH2 | 1 | Inhibits |
| AKT2 | FOXO1 | 2,3 | Inhibits |
| AKT2 | MTOR | 4 | Promotes |
| AKT2 | PPARGC1A | 5 | Inhibits |
| AKT2 | SLC2A1 | 6 | Promotes |
| AKT2 | SLC2A2 | 6 | Promotes |
| AKT2 | SLC2A3 | 6 | Promotes |
| AKT2 | SLC2A4 | 6 | Promotes |
| AKT2 | SLC2A5 | 6 | Promotes |
| AKT3 | BACH2 | 1 | Inhibits |
| AKT3 | FOXO1 | 2,3 | Inhibits |
| AKT3 | MTOR | 4 | Promotes |
| AKT3 | PPARGC1A | 5 | Inhibits |
| AKT3 | SLC2A1 | 6 | Promotes |
| AKT3 | SLC2A2 | 6 | Promotes |
| AKT3 | SLC2A3 | 6 | Promotes |
| AKT3 | SLC2A4 | 6 | Promotes |
| AKT3 | SLC2A5 | 6 | Promotes |
| BACH2 | GATA3 | 7 | Inhibits |
| BACH2 | ID3 | 7 | Inhibits |
| BACH2 | JUN | 7 | Inhibits |
| BACH2 | JUNB | 1,7 | Inhibits |
| BACH2 | JUND | 1,7 | Inhibits |
| BACH2 | PRDM1 | 7,8 | Inhibits |
| BATF | BCL6 | 9 | Promotes |
| BATF | PRDM1 | 10 | Promotes |
| BATF | EOMES | 11 | Promotes |
| BATF | IRF4 | 11 | Inhibits |
| BATF | NFATC2 | 11 | Promotes |
| BATF | NF-κB1 | 11 | Promotes |
| BATF | RUNX3 | 11 | Promotes |
| BATF | TBX21 | 11 | Promotes |
| BCL6 | GZMB | 12 | Inhibits |
| BCL6 | ID2 | 13 | Inhibits |
| BCL6 | LEF1 | 13 | Promotes |
| BCL6 | PRDM1 | 13,14 | Inhibits |
| BCL6 | TCF7 | 13 | Promotes |
| BTLA | AKT1 | 15 | Inhibits |
| BTLA | AKT2 | 15 | Inhibits |
| BTLA | AKT3 | 15 | Inhibits |
| BTLA | NF-κB1 | 15 | Inhibits |
| BTLA | NF-κB2 | 15 | Inhibits |
| BTLA | PIK3CA | 15 | Inhibits |
| BTLA | PIK3CB | 15 | Inhibits |
| BTLA | PIK3CG | 15 | Inhibits |
| BTLA | PRKCQ | 15 | Inhibits |
| BTLA | REL | 15 | Promotes |
| BTLA | RELA | 15 | Inhibits |
| BTLA | RELB | 15 | Inhibits |
| CA | NFATC1 | 16 | Promotes |
| CA | NFATC2 | 16 | Promotes |
| CD160 | IFN-γ | 17 | Inhibits |
| CD160 | IL2 | 17 | Inhibits |
| CD244 | CA | 18 | Promotes |
| CD244 | MAPK1 | 18 | Promotes |
| CD244 | MAPK3 | 18 | Promotes |
| CD244 | NFATC1 | 18 | Promotes |
| CD244 | NFATC2 | 18 | Promotes |
| CD244 | NF-κB1 | 19 | Promotes |
| CD244 | NF-κB2 | 19 | Promotes |
| CD244 | REL | 19 | Promotes |
| CD244 | RELA | 19 | Promotes |
| CD244 | RELB | 19 | Promotes |
| CD247 | AKT1 | 20 | Promotes |
| CD247 | AKT2 | 20 | Promotes |
| CD247 | AKT3 | 20 | Promotes |
| CD247 | DNMT3A | 21 | Promotes |
| CD247 | FOS | 22 | Promotes |
| CD247 | JUN | 22 | Promotes |
| CD247 | JUNB | 22 | Promotes |
| CD247 | JUND | 22 | Promotes |
| CD247 | NFATC1 | 16 | Promotes |
| CD247 | NFATC2 | 16 | Promotes |
| CD247 | NF-κB1 | 23 | Promotes |
| CD247 | NF-κB2 | 23 | Promotes |
| CD247 | REL | 23 | Promotes |
| CD247 | RELA | 23 | Promotes |
| CD247 | RELB | 23 | Promotes |
| CD247 | ZAP70 | 24 | Promotes |
| CD274 | ZAP70 | 25 | Inhibits |
| CD28 | DNMT3A | 21 | Promotes |
| CD28 | HRAS | 15 | Promotes |
| CD28 | KRAS | 15 | Promotes |
| CD28 | NF-κB1 | 26 | Promotes |
| CD28 | NRAS | 15 | Promotes |
| CD28 | PIK3CA | 26 | Promotes |
| CD28 | PIK3CB | 26 | Promotes |
| CD28 | PIK3CG | 26 | Promotes |
| CD28 | PRKCQ | 26,27 | Promotes |
| CD28 | RELA | 26 | Promotes |
| CD3D | AKT1 | 20 | Promotes |
| CD3D | AKT2 | 20 | Promotes |
| CD3D | AKT3 | 20 | Promotes |
| CD3D | DNMT3A | 21 | Promotes |
| CD3D | FOS | 22 | Promotes |
| CD3D | JUN | 22 | Promotes |
| CD3D | JUNB | 22 | Promotes |
| CD3D | JUND | 22 | Promotes |
| CD3D | NFATC1 | 16 | Promotes |
| CD3D | NFATC2 | 16 | Promotes |
| CD3D | NF-κB1 | 23 | Promotes |
| CD3D | NF-κB2 | 23 | Promotes |
| CD3D | REL | 23 | Promotes |
| CD3D | RELA | 23 | Promotes |
| CD3D | RELB | 23 | Promotes |
| CD3D | ZAP70 | 24 | Promotes |
| CD3E | AKT1 | 20 | Promotes |
| CD3E | AKT2 | 20 | Promotes |
| CD3E | AKT3 | 20 | Promotes |
| CD3E | DNMT3A | 21 | Promotes |
| CD3E | FOS | 22 | Promotes |
| CD3E | JUN | 22 | Promotes |
| CD3E | JUNB | 22 | Promotes |
| CD3E | JUND | 22 | Promotes |
| CD3E | NFATC1 | 16 | Promotes |
| CD3E | NFATC2 | 16 | Promotes |
| CD3E | NF-κB1 | 23 | Promotes |
| CD3E | NF-κB2 | 23 | Promotes |
| CD3E | REL | 23 | Promotes |
| CD3E | RELA | 23 | Promotes |
| CD3E | RELB | 23 | Promotes |
| CD3E | ZAP70 | 24 | Promotes |
| CD3G | AKT1 | 20 | Promotes |
| CD3G | AKT2 | 20 | Promotes |
| CD3G | AKT3 | 20 | Promotes |
| CD3G | DNMT3A | 21 | Promotes |
| CD3G | FOS | 22 | Promotes |
| CD3G | JUN | 22 | Promotes |
| CD3G | JUNB | 22 | Promotes |
| CD3G | JUND | 22 | Promotes |
| CD3G | NFATC1 | 16 | Promotes |
| CD3G | NFATC2 | 16 | Promotes |
| CD3G | NF-κB1 | 23 | Promotes |
| CD3G | NF-κB2 | 23 | Promotes |
| CD3G | REL | 23 | Promotes |
| CD3G | RELA | 23 | Promotes |
| CD3G | RELB | 23 | Promotes |
| CD3G | ZAP70 | 24 | Promotes |
| CD8A | CD247 | 28 | Promotes |
| CD8A | CD3D | 28 | Promotes |
| CD8A | CD3E | 28 | Promotes |
| CD8A | CD3G | 28 | Promotes |
| CD8A | DNMT3A | 21 | Promotes |
| CD8A | LCK | 26 | Promotes |
| CTLA4 | AKT1 | 15 | Inhibits |
| CTLA4 | AKT2 | 15 | Inhibits |
| CTLA4 | AKT3 | 15 | Inhibits |
| CTLA4 | NF-κB1 | 15 | Inhibits |
| CTLA4 | NF-κB2 | 15 | Inhibits |
| CTLA4 | REL | 15 | Inhibits |
| CTLA4 | RELA | 15 | Inhibits |
| CTLA4 | RELB | 15 | Inhibits |
| DNMT3A | DNMT3A | 29 | Inhibits |
| DNMT3A | TCF7 | 30,31 | Inhibits |
| DNMT3B | DNMT3A | 29 | Inhibits |
| DNMT3L | DNMT3A | 29 | Inhibits |
| EGR1 | ID3 | 32 | Promotes |
| EGR1 | IL2 | 33 | Promotes |
| EGR1 | IL2RB | 34 | Promotes |
| EGR2 | GZMB | 35 | Inhibits |
| EGR2 | ID3 | 35 | Promotes |
| EGR2 | IFN-γ | 35 | Inhibits |
| EGR2 | LAG3 | 36 | Promotes |
| EGR2 | MYC | 35 | Promotes |
| EGR2 | PRDM1 | 35 | Inhibits |
| EGR2 | TBX21 | 35 | Inhibits |
| EGR2 | TCF7 | 35 | Promotes |
| EGR2 | ZEB2 | 35 | Promotes |
| EGR3 | GZMB | 35 | Inhibits |
| EGR3 | ID3 | 35 | Promotes |
| EGR3 | IFN-γ | 35 | Inhibits |
| EGR3 | MYC | 35 | Promotes |
| EGR3 | PRDM1 | 35 | Inhibits |
| EGR3 | TBX21 | 35 | Inhibits |
| EGR3 | TCF7 | 35 | Promotes |
| EGR3 | ZEB2 | 35 | Promotes |
| Exhaustion | ZN | 37 | Promotes |
| EZH2 | DNMT3A | 38 | Promotes |
| EZH2 | DNMT3B | 38 | Promotes |
| EZH2 | DNMT3L | 38 | Promotes |
| FOS | IFN-γ | 39 | Promotes |
| FOS | IL2 | 40 | Promotes |
| FOS | IL2RB | 41 | Promotes |
| FOS | PDCD1 | 42 | Promotes |
| FOS | PRDM1 | 14,40 | Promotes |
| FOXO1 | BCL6 | 43 | Promotes |
| FOXO1 | EZH2 | 44 | Inhibits |
| FOXO1 | PDCD1 | 45,46 | Promotes |
| GATA3 | BATF | 11 | Promotes |
| GATA3 | MYC | 47 | Promotes |
| HIF1A | BATF | 11 | Promotes |
| HIF1A | MYC | 48 | Promotes |
| HRAS | MAPK1 | 49 | Promotes |
| HRAS | MAPK3 | 49 | Promotes |
| HRAS | MAPK8 | 49 | Promotes |
| ICOS | MAF | 15 | Promotes |
| ID2 | TCF3 | 13 | Inhibits |
| ID3 | TCF3 | 13 | Inhibits |
| IFNAR1 | BCL6 | 50,51 | Promotes |
| IFNAR1 | MT1 | 52 | Promotes |
| IFNAR1 | MT2 | 52 | Promotes |
| IFNAR1 | PDCD1 | 42 | Promotes |
| IFNAR1 | PIK3CA | 53 | Promotes |
| IFNAR1 | PIK3CB | 53 | Promotes |
| IFNAR1 | PIK3CG | 53 | Promotes |
| IFNAR1 | PRKCQ | 53 | Promotes |
| IFNAR1 | STAT1 | 54 | Promotes |
| IFNAR1 | STAT4 | 55 | Promotes |
| IFNAR2 | BCL6 | 50,51 | Promotes |
| IFNAR2 | MT1 | 52 | Promotes |
| IFNAR2 | MT2 | 52 | Promotes |
| IFNAR2 | PDCD1 | 42 | Promotes |
| IFNAR2 | PIK3CA | 53 | Promotes |
| IFNAR2 | PIK3CB | 53 | Promotes |
| IFNAR2 | PIK3CG | 53 | Promotes |
| IFNAR2 | PRKCQ | 53 | Promotes |
| IFNAR2 | STAT1 | 54 | Promotes |
| IFNAR2 | STAT4 | 55 | Promotes |
| IFN-γ | EGR2 | 35 | Inhibits |
| IFN-γ | EGR3 | 35 | Inhibits |
| IFN-γ | MT1 | 52 | Promotes |
| IFN-γ | MT2 | 52 | Promotes |
| IFN-γ | PIK3CA | 53 | Promotes |
| IFN-γ | PIK3CB | 53 | Promotes |
| IFN-γ | PIK3CG | 53 | Promotes |
| IFN-γ | PRKCQ | 53 | Promotes |
| IFN-γ | SLC2A1 | 56 | Inhibits |
| IFN-γ | SLC2A2 | 56 | Inhibits |
| IFN-γ | SLC2A3 | 56 | Inhibits |
| IFN-γ | SLC2A4 | 56 | Inhibits |
| IFN-γ | SLC2A5 | 56 | Inhibits |
| IFN-γ | STAT1 | 57 | Inhibits |
| IFN-γ | STAT2 | 57 | Inhibits |
| IFNGR1 | EGR2 | 35 | Inhibits |
| IFNGR1 | EGR3 | 35 | Inhibits |
| IFNGR1 | MT2 | 52 | Promotes |
| IFNGR1 | PIK3CA | 53 | Promotes |
| IFNGR1 | PIK3CB | 53 | Promotes |
| IFNGR1 | PIK3CG | 53 | Promotes |
| IFNGR1 | PRKCQ | 53 | Promotes |
| IFNGR2 | EGR2 | 35 | Inhibits |
| IFNGR2 | EGR3 | 35 | Inhibits |
| IFNGR2 | MT2 | 52 | Promotes |
| IFNGR2 | PIK3CA | 53 | Promotes |
| IFNGR2 | PIK3CB | 53 | Promotes |
| IFNGR2 | PIK3CG | 53 | Promotes |
| IFNGR2 | PRKCQ | 53 | Promotes |
| IL12RB1 | BATF | 58 | Promotes |
| IL12RB1 | ID2 | 59 | Promotes |
| IL12RB1 | PRDM1 | 59 | Promotes |
| IL12RB1 | STAT4 | 60,61 | Promotes |
| IL12RB1 | TBX21 | 62 | Promotes |
| IL12RB2 | BATF | 58 | Promotes |
| IL12RB2 | ID2 | 59 | Promotes |
| IL12RB2 | PRDM1 | 59 | Promotes |
| IL12RB2 | STAT4 | 60,61 | Promotes |
| IL12RB2 | TBX21 | 62 | Promotes |
| IL2 | BCL6 | 41 | Inhibits |
| IL2 | IL2RB | 41 | Promotes |
| IL21R | BATF | 10 | Promotes |
| IL21R | STAT3 | 63,64 | Promotes |
| IL2RA | BCL6 | 43,65 | Inhibits |
| IL2RA | FOXO1 | 43,66 | Inhibits |
| IL2RA | GATA3 | 67 | Promotes |
| IL2RA | HIF1A | 65 | Promotes |
| IL2RA | ID2 | 59 | Promotes |
| IL2RA | PRDM1 | 59 | Promotes |
| IL2RB | BCL6 | 43,65 | Inhibits |
| IL2RB | CD160 | 68 | Promotes |
| IL2RB | CD244 | 68 | Promotes |
| IL2RB | FOXO1 | 43,66 | Inhibits |
| IL2RB | GATA3 | 67 | Promotes |
| IL2RB | HAVCR2 | 68 | Promotes |
| IL2RB | HIF1A | 65 | Promotes |
| IL2RB | ID2 | 59 | Promotes |
| IL2RB | LAG3 | 68 | Promotes |
| IL2RB | MYC | 68 | Promotes |
| IL2RB | PDCD1 | 68 | Promotes |
| IL2RB | PRDM1 | 59 | Promotes |
| IRF4 | BCL6 | 10,69 | Inhibits |
| IRF4 | PRDM1 | 10,69 | Promotes |
| JUN | IFN-γ | 39 | Promotes |
| JUN | IFN-γ R1 | 39 | Promotes |
| JUN | IFN-γ R2 | 39 | Promotes |
| JUN | IL2 | 40 | Promotes |
| JUN | IL2RB | 41 | Promotes |
| JUN | PDCD1 | 42 | Promotes |
| JUN | PRDM1 | 14,40 | Promotes |
| JUNB | IFN-γ | 39 | Promotes |
| JUNB | IL2 | 40 | Promotes |
| JUNB | IL2RB | 41 | promotes |
| JUNB | PDCD1 | 42 | Promotes |
| JUNB | PRDM1 | 40 | Promotes |
| JUND | IFN-γ | 39 | Promotes |
| JUND | IL2 | 40 | Promotes |
| JUND | IL2RB | 41 | Promotes |
| JUND | PDCD1 | 42 | Promotes |
| JUND | PRDM1 | 40 | Promotes |
| KDM6B | EZH2 | 70 | Inhibits |
| KRAS | MAPK1 | 49 | Promotes |
| KRAS | MAPK3 | 49 | Promotes |
| KRAS | MAPK8 | 49 | Promotes |
| LCK | ZAP70 | 26 | Promotes |
| LEF1 | BCL6 | 71 | Promotes |
| LEF1 | CXCR5 | 72 | Promotes |
| LEF1 | EOMES | 73 | Promotes |
| LEF1 | GZMA | 73 | Inhibits |
| LEF1 | GZMB | 73 | Inhibits |
| LEF1 | IFN-γ | 74 | Inhibits |
| LEF1 | KLRG1 | 73 | Inhibits |
| LEF1 | LEF1 | 13 | Promotes |
| LEF1 | MYC | 73 | Promotes |
| LEF1 | PRDM1 | 13 | Promotes |
| LEF1 | PRF1 | 75 | Inhibits |
| MAPK1 | EGR1 | 76 | Promotes |
| MAPK1 | FOS | 26 | Promotes |
| MAPK1 | JUN | 26 | Promotes |
| MAPK1 | JUNB | 26 | Promotes |
| MAPK1 | JUND | 26 | Promotes |
| MAPK3 | EGR1 | 76 | Promotes |
| MAPK3 | FOS | 26 | Promotes |
| MAPK3 | JUN | 26 | Promotes |
| MAPK3 | JUNB | 26 | Promotes |
| MAPK3 | JUND | 26 | Promotes |
| MAPK8 | FOS | 26 | Promotes |
| MAPK8 | JUN | 26 | Promotes |
| MAPK8 | JUNB | 26 | Promotes |
| MAPK8 | JUND | 26 | Promotes |
| MT1 | MAPK1 | 77 | Promotes |
| MT1 | MAPK3 | 77 | Promotes |
| MT1 | ZN | 37 | Promotes |
| MT2 | ZN | 37 | Promotes |
| MTOR | Glycolysis | 4 | Promotes |
| MTOR | RICTOR | 78 | Promotes |
| MTOR | RPTOR | 78 | Promotes |
| MYC | Proliferation | 48 | Promotes |
| NFATC1 | IFN-γ | 39,79 | Promotes |
| NFATC1 | IFNGR1 | 39 | Promotes |
| NFATC1 | IFNGR2 | 39 | Promotes |
| NFATC1 | IL2 | 40 | Promotes |
| NFATC1 | IL2RB | 41 | Promotes |
| NFATC1 | NFATC1 | 80 | Promotes |
| NFATC1 | NR4A1 | 81 | Promotes |
| NFATC1 | PDCD1 | 42 | Promotes |
| NFATC1 | PRDM1 | 40 | Promotes |
| NFATC2 | EGR2 | 36 | Promotes |
| NFATC2 | EGR3 | 35 | Promotes |
| NFATC2 | IFN-γ | 39,79 | Promotes |
| NFATC2 | IL2 | 40 | Promotes |
| NFATC2 | IL2RB | 41 | Promotes |
| NFATC2 | NFATC1 | 82 | Promotes |
| NFATC2 | PDCD1 | 42 | Promotes |
| NFATC2 | PRDM1 | 40 | Promotes |
| NF-κB1 | IL2 | 40 | Promotes |
| NF-κB1 | IL2RB | 41 | Promotes |
| NF-κB1 | IRF4 | 83 | Promotes |
| NF-κB1 | PDCD1 | 42 | Promotes |
| NF-κB1 | PRDM1 | 40 | Promotes |
| NF-κB2 | IRF4 | 83 | Promotes |
| NR4A1 | Glycolysis | 84 | Promotes |
| NR4A1 | IRF4 | 85 | Inhibits |
| NR4A2 | Glycolysis | 86 | Promotes |
| NR4A3 | Glycolysis | 87 | Promotes |
| NRAS | MAPK1 | 49 | Promotes |
| NRAS | MAPK3 | 49 | Promotes |
| NRAS | MAPK8 | 49 | Promotes |
| PDCD1 | AKT1 | 88 | Promotes |
| PDCD1 | AKT2 | 88 | Promotes |
| PDCD1 | AKT3 | 88 | Promotes |
| PDCD1 | CD247 | 89 | Inhibits |
| PDCD1 | CD28 | 89 | Inhibits |
| PDCD1 | CD3D | 89 | Inhibits |
| PDCD1 | CD3E | 89 | Inhibits |
| PDCD1 | CD3G | 89 | Inhibits |
| PDCD1 | CD8A | 89 | Inhibits |
| PDCD1 | NR4A1 | 86 | Inhibits |
| PDCD1 | BATF | 90 | Promotes |
| PDCD1 | HRAS | 91 | Inhibits |
| PDCD1 | KRAS | 91 | Inhibits |
| PDCD1 | NRAS | 91 | Inhibits |
| PDCD1 | PIK3CA | 91 | Inhibits |
| PDCD1 | PIK3CB | 91 | Inhibits |
| PDCD1 | PIK3CG | 91 | Inhibits |
| PDCD1 | PPARGC1A | 88 | Inhibits |
| PDCD1 | PRKCQ | 15 | Inhibits |
| PDCD1 | SLC2A1 | 56 | Promotes |
| PDCD1 | SLC2A2 | 56 | Promotes |
| PDCD1 | SLC2A3 | 56 | Promotes |
| PDCD1 | SLC2A4 | 56 | Promotes |
| PDCD1 | SLC2A5 | 56 | Promotes |
| PDCD1 | ZAP70 | 25 | Inhibits |
| PPARGC1A | Proliferation | 5 | Promotes |
| PRDM1 | BCL6 | 13,92,93 | Inhibits |
| PRDM1 | CXCR5 | 13 | Inhibits |
| PRDM1 | ID3 | 94 | Inhibits |
| PRDM1 | IL2 | 94 | Inhibits |
| PRDM1 | MYC | 95 | Inhibits |
| PRDM1 | NFATC1 | 96 | Inhibits |
| PRDM1 | PDCD1 | 96 | Inhibits |
| PRDM1 | TCF7 | 13 | Inhibits |
| PRKCQ | NF-κB1 | 97 | Promotes |
| PRKCQ | RELA | 97 | Promotes |
| REL | IRF4 | 83 | Promotes |
| RELA | IL2 | 40 | Promotes |
| RELA | IL2RB | 41 | Promotes |
| RELA | IRF4 | 83 | Promotes |
| RELA | PDCD1 | 42 | Promotes |
| RELA | PRDM1 | 40 | Promotes |
| RELB | IRF4 | 83 | Promotes |
| RUNX3 | BATF | 11 | Promotes |
| RUNX3 | IFN-γ | 98 | Promotes |
| SLC39A14 | ZN | 99 | Promotes |
| SLC39A8 | ZN | 99 | Promotes |
| STAT1 | BCL6 | 50,51 | Promotes |
| STAT1 | CXCR5 | 50,51 | Inhibits |
| STAT1 | PDCD1 | 50,51 | Promotes |
| STAT2 | BCL6 | 50,51 | Promotes |
| STAT3 | BATF | 11 | Promotes |
| STAT3 | FOXO1 | 100 | Promotes |
| STAT4 | IFN-γ | 55 | Promotes |
| STAT4 | IFNGR1 | 55 | Promotes |
| STAT4 | IFNGR2 | 55 | Promotes |
| STAT4 | STAT1 | 101 | Inhibits |
| STAT4 | TBX21 | 55 | Promotes |
| TBX21 | BCL6 | 102 | Inhibits |
| TBX21 | BTLA | 103 | Inhibits |
| TBX21 | CD160 | 103 | Inhibits |
| TBX21 | HAVCR2 | 104 | Promotes |
| TBX21 | IFN-γ | 98 | Promotes |
| TBX21 | IL2RB | 105 | Promotes |
| TBX21 | LAG3 | 103 | Inhibits |
| TBX21 | PDCD1 | 103 | Inhibits |
| TBX21 | RUNX3 | 98 | Promotes |
| TBX21 | ZEB2 | 106 | Promotes |
| TCF3 | CXCR5 | 13 | Promotes |
| TCF3 | TCF7 | 13 | Promotes |
| TCF7 | BCL6 | 71 | Promotes |
| TCF7 | CXCR5 | 72 | Promotes |
| TCF7 | EOMES | 73 | Promotes |
| TCF7 | GATA3 | 74 | Promotes |
| TCF7 | GZMA | 73 | Inhibits |
| TCF7 | GZMB | 73 | Inhibits |
| TCF7 | IFN-γ | 74 | Inhibits |
| TCF7 | IFNGR1 | 74 | Inhibits |
| TCF7 | IFNGR2 | 74 | Inhibits |
| TCF7 | KLRG1 | 73 | Inhibits |
| TCF7 | MYC | 73 | Promotes |
| TCF7 | PRDM1 | 13 | Inhibits |
| TCF7 | PRF1 | 75 | Inhibits |
| TET1 | DNMT3A | 107,108 | Inhibits |
| TET1 | DNMT3B | 107,108 | Inhibits |
| TET1 | DNMT3L | 107,108 | Inhibits |
| TET1 | PDCD1 | 109 | Promotes |
| TET2 | DNMT3A | 107,108 | Inhibits |
| TET2 | DNMT3B | 107,108 | Inhibits |
| TET2 | DNMT3L | 107,108 | Inhibits |
| TET2 | PDCD1 | 109 | Promotes |
| TET3 | DNMT3A | 107,108 | Inhibits |
| TET3 | DNMT3B | 107,108 | Inhibits |
| TET3 | DNMT3L | 107,108 | Inhibits |
| TET3 | PDCD1 | 109 | Promotes |
| TIGIT | MAPK1 | 110 | Inhibits |
| TIGIT | MAPK3 | 110 | Inhibits |
| TIGIT | NF-κB1 | 110 | Inhibits |
| TIGIT | NF-κB2 | 110 | Inhibits |
| TIGIT | PIK3CA | 110 | Inhibits |
| TIGIT | PIK3CB | 110 | Inhibits |
| TIGIT | PIK3CG | 110 | Inhibits |
| TIGIT | REL | 110 | Inhibits |
| TIGIT | RELA | 110 | Inhibits |
| TIGIT | RELB | 110 | Inhibits |
| TNF | NF-κB1 | 111 | Promotes |
| TNF | NF-κB2 | 111 | Promotes |
| TNF | REL | 111 | Promotes |
| TNF | RELA | 111 | Promotes |
| TNF | RELB | 111 | Promotes |
| TNFRSF1A | TNF | 111 | Promotes |
| TNFRSF1B | TNF | 111 | Promotes |
| TNFRSF9 | CD247 | 112 | Promotes |
| TNFRSF9 | CD28 | 112 | Promotes |
| TNFRSF9 | CD3D | 112 | Promotes |
| TNFRSF9 | CD3E | 112 | Promotes |
| TNFRSF9 | CD3G | 112 | Promotes |
| TNFRSF9 | CD8A | 112 | Promotes |
| TNFRSF9 | FOXO1 | 113 | Inhibits |
| ZAP70 | Adhesion | 24 | Promotes |
| ZAP70 | CA | 24 | Promotes |
| ZAP70 | HRAS | 24 | Promotes |
| ZAP70 | KRAS | 24 | Promotes |
| ZAP70 | NRAS | 24 | Promotes |
| ZAP70 | PRKCQ | 26,27 | Promotes |
| ZEB1 | IL2 | 114 | Inhibits |
| ZEB2 | IL2 | 114 | Inhibits |
| ZEB2 | TBX21 | 115 | Promotes |
| ZN | MT1 | 77 | Promotes |
| ZN | MT2 | 77 | Promotes |

# **Supplementary Table S2. Reduced TCE network specifications**

| **Source** | **Target** | **Interaction** | **Reference** | **Comment** |
| --- | --- | --- | --- | --- |
| 4-1BB | FAS/FASL | Promotes | 116,117 | Delayed, peaks at ~3 days |
| 4-1BB | TCF-1 | Promotes | 113 |  |
| AKT | BACH2 | Promotes | 1 |  |
| AKT | NFATC1,2 | Promotes | 118-120 | NFATs are activated by Ca^++^ signaling. AKT stops nuclear export of activated NFATs. |
| AKT | MTOR | Promotes | 121,122 |  |
| AKT | NF-κB | Promotes | 123 |  |
| AKT | FOXO1 | Inhibits | 2 |  |
| AKT | AP1 | Promotes | 124 |  |
| AP1 (JUN:FOS) | NR4A1 | Promotes | 125,126 | AP1 causes NR4A1 nuclear export, which activates its pro-apoptotic activity in mitochondria. See also ^127,128^ |
| AP1 (JUN:FOS) | NFATC1:AP1:IRF4:BATF | Inhibits | 129-132 | BATF & AP1 compete for the same sites^131^ |
| BACH2 | IFN-γ | Inhibits | 1 |  |
| BACH2 | BLIMP-1 | Inhibits | 133 |  |
| BACH2 | ID3 | Promotes | 134 |  |
| BATF | NFATC1:AP1:IRF4:BATF | Promotes | 10,130,132,135 | See also ^136^ |
| BCL6 | TCF-1 | Promotes | 13,71,72 |  |
| BCL6 | BLIMP-1 | Inhibits | 137 |  |
| BLIMP-1 | TIGIT | Promotes | 138 |  |
| BLIMP-1 | CD160 | Promotes | 139,140 |  |
| BLIMP-1 | CD244/2B4 | Promotes | 139,140 |  |
| BLIMP-1 | BCL6 | Inhibits | 141 |  |
| BLIMP-1 | ID3 | Inhibits | 142 |  |
| BTLA | CD3, TCR, CD8, CD28 | Inhibits | 143 |  |
| CD137L | 4-1BB | Promotes | 144 |  |
| CD3, TCR, CD8, CD28 | 4-1BB | Promotes | 145 |  |
| CD3, TCR, CD8, CD28 | RAS | Promotes | 15,146 |  |
| CD3, TCR, CD8, CD28 | EZH2/PRC2 | Promotes | 147 | PRC2 has a dual role^148^ |
| CD3, TCR, CD8, CD28 | PI3K | Promotes | 15 |  |
| CTLA4 | Tregs | Promotes | 149 |  |
| CTLA4 | CD3, TCR, CD8, CD28 | Inhibits | 149 |  |
| E2A | CXCR5 | Promotes | 150 |  |
| EGR2/3 | TBET:ZEB2 | Inhibits | 35,151 |  |
| EGR2/3 | LAG3 | Promotes | 152 |  |
| EGR2/3 | TCF-1 | Promotes | 35 |  |
| EGR2/3 | ID3 | Promotes | 35 |  |
| EGRs | FAS/FASL | Promotes | 153,154 |  |
| EOMES | GZMA/B | Promotes | 155 |  |
| EOMES | PRF1 | Promotes | 155 |  |
| EOMES | IFN-γ | Promotes | 155 |  |
| EZH2/PRC2 | EOMES | Inhibits | 44 |  |
| EZH2/PRC2 | TCF-1 | Inhibits | 44 |  |
| EZH2/PRC2 | BACH2 | Inhibits | 44 |  |
| EZH2/PRC2 | FOXO1 | Inhibits | 44 |  |
| FAS/FASL | FAS/FASL | Promotes | 156 |  |
| FOXO1 | PGC1A | Promotes | 5,157-160 |  |
| FOXO1 | TCF-1 | Promotes | 161 |  |
| HIF1A | MYC | Promotes | 48 |  |
| HIF1A | PPARa | Inhibits | 162,163 |  |
| ID3 | FAS/FASL | Inhibits | 164 |  |
| ID3 | IL2R | Inhibits | 165 |  |
| ID3:Tnaïve | E2A action on CXCR5 | Inhibits | 166 |  |
| ID3:Tstim | CXCR5 | Promotes | 165 | See their Fig. 4e, f |
| IFN-γ | FAS/FASL | Promotes | 167 | IFN-γ signaling is ***required*** for FASL expression |
| IL12R | TBET:ZEB2 | Promotes | 168,169 |  |
| IL12R | ID2 | Promotes | 165 |  |
| IL12R | BATF | Promotes | 58 |  |
| IL12R | FOXO1 | Inhibits | 64 |  |
| IL15R | TIGIT | Promotes | 170 |  |
| IL21R | ID2 | Promotes | 165 |  |
| IL21R | IRF4 | Promotes | 10 |  |
| IL21R | TBET:ZEB2 | Promotes | 171 |  |
| IL21R | BATF | Promotes | 10 |  |
| IL2R | TIGIT | Promotes | 170 |  |
| IL2R | BLIMP-1 | Promotes | 59 |  |
| IL2R | FOXO1 | Inhibits | 172 | Possible mutual repression^100^ |
| IL2R | IRF4 | Promotes | 173 |  |
| IL2R | ID2 | Promotes | 165 |  |
| IRF4 | NFATC1:AP1:IRF4:BATF | Promotes | 10,132 |  |
| LAG3 | Tregs | Promotes | 174 |  |
| MAPK | EGR2/3 | Promotes | 76 | See their Fig. 2 |
| MAPK | AP1 | Promotes | 175 |  |
| MTOR | MYC | Promotes | 176 | Reviewed in ^177^ |
| MTOR | HIF1A | Promotes | 176 | Reviewed in ^177^. HIF1A protein reaches peak levels by 24hr p.i.^178^.  At later time points (~3 days p.i.^179^), HIF1A is jointly activated by IRF4 + BATF + NFAT^135^. |
| MTOR | IRF4 | Promotes | 179 | Reviewed in ^177^ |
| MYC | Glycolysis | Promotes | 180 | Reviewed in ^177^ |
| MYC | Proliferation | Promotes | 180 | Reviewed in ^177^ |
| MYC | MT biogenesis | Promotes | 181,182 |  |
| MYC | FAS/FASL | Promotes | 154,183,184 |  |
| NFAT | FAS/FASL | Promotes | 154,185 |  |
| NFATC1 | TIM3 | Promotes | 36 | NFATC1 and NFATC2 bind the same motif^186^ |
| NFATC1 | LAG3 | Promotes | 36 | NFATC1 and NFATC2 bind the same motif^186^ |
| NFATC1 | CTLA4 | Promotes | 36 | NFATC1 and NFATC2 bind the same motif^186^ |
| NFATC1 | PD-1 | Promotes | 36,187 | NFATC1 and NFATC2 bind the same motif^186^ |
| NFATC1 | NFATC1 | Promotes | 80,188 | NFATC1 and NFATC2 bind the same motif^186^ |
| NFATC1 | IRF4 | Promotes | 135 | NFATC1 and NFATC2 bind the same motif^186^ |
| NFATC1 | NFATC1:AP1:IRF4:BATF | Promotes | 135 |  |
| NFATC1:AP1:IRF4:BATF | MYC | Inhibits | 189 |  |
| NFATC1:AP1:IRF4:BATF | NFATC1 | Promotes | 135 |  |
| NFATC1:AP1:IRF4:BATF | BCL6 | Inhibits | 69 |  |
| NFATC2 | CTLA4 | Promotes | 190 | NFATC1 and NFATC2 bind the same motif^186^ |
| NFATC2 | PD-1 | Promotes | 36 |  |
| NFATC2 | NFATC1 | Promotes | 80 | See also ^191^ |
| NFATC2 | IL12R | Inhibits | 192 |  |
| NFATs | BCL6 | Promotes | 193 | Indirect, via Bob1 |
| NF-κB | BTLA | Promotes | 193 | Indirect, via Bob1 |
| NF-κB | BCL6 | Promotes | 193 | Indirect, via Bob1 |
| NR4A1 | FAS/FASL | Promotes | 144 |  |
| PD-1 | CD3, TCR, CD8, CD28 | Inhibits | 89,194 |  |
| PGC1A | MT biogenesis | Promotes | 5,195,196 |  |
| PI3K | AKT | Promotes | 197 |  |
| RAS | MAPK | Promotes | 198 |  |
| RUNX3 | GZMA/B | Promotes | 155 |  |
| RUNX3 | PRF1 | Promotes | 155 |  |
| RUNX3 | IFN-γ | Promotes | 155 |  |
| RUNX3 | EOMES | Promotes | 155 |  |
| TBET | ZEB2 | Promotes | 115 |  |
| TBET:ZEB2 | GZMA/B | Promotes | 115 |  |
| TBET:ZEB2 | IFN-γ | Promotes | 115 |  |
| TBET:ZEB2 | PD-1 | Inhibits | 103,115 |  |
| TCF-1 | KLRG1 | Inhibits | 73 |  |
| TCF-1 | BCL6 | Promotes | 199,200 |  |
| TIGIT | degranulation | Inhibits | 201 |  |
| TIGIT | Tregs | Promotes | 202 |  |
| TIM3 | CD3, TCR, CD8, CD28 | Inhibits | 203 |  |
| ZEB2 | ID3 | Inhibits | 115 | Repression may be mutual^165^ |
| ZEB2 | TBET | Promotes | 115 |  |

### **Supplementary Table S3. Timings of TCE network interactions**

| **Source** | **Target** | **Interaction** | **Reference** | **Comment** |
| --- | --- | --- | --- | --- |
| 4-1BB | FAS/FASL | Promotes | 204,205 | 4-1BB expression is delayed, peaking at about 1-5 days p.i.^116,117^, and its action on FAS signaling is gated by IFN-γ^167^. |
| AKT | FOXO1 | Inhibits | 64,206 | FOXO1 protein is high in naïve cells and down-regulated over 2–3 days post stim. |
| EZH2 | FOXO1, TCF-1 | Inhibits | 207 | EZH2 expression peaks at 1 day post stim in CD8+ cells |
| EZH2 | TCF-1 | Inhibits | 44 | H3K27me3 of TCF-1 locus peaks at ~ 10 days post-infection. |
| DNMT3A | Downstream of EZH2 activity | Inhibits | 21 | DNMT3A is activated by TCR stim. and peaks at ~24 hours. |
| DNMTs | TCF-1 | Inhibits | 30,208 | TCF-1 locus is methylated after day 4 and by ~ day 8 post-infection. |

## Supplementary References (for the Supplementary Tables)

1. Roychoudhuri, R. et al. BACH2 regulates CD8^+^ T cell differentiation by controlling access of AP-1 factors to enhancers. *Nat. Immunol.* **17,** 851-860 (2016).

2. Calnan, D. R. & Brunet, A. The FoxO code. *Oncogene* **27,** 2276-2288 (2008).

3. Hedrick, S. M., Hess Michelini, R.,  [Doedens, A. L](https://www.ncbi.nlm.nih.gov/pubmed/?term=Doedens%20AL%5BAuthor%5D&cauthor=true&cauthor_uid=22918467)., [Goldrath, A. W](https://www.ncbi.nlm.nih.gov/pubmed/?term=Goldrath%20AW%5BAuthor%5D&cauthor=true&cauthor_uid=22918467). & [Stone E. L](https://www.ncbi.nlm.nih.gov/pubmed/?term=Stone%20EL%5BAuthor%5D&cauthor=true&cauthor_uid=22918467). FOXO transcription factors throughout T cell biology. *Nat. Rev. Immunol.* **12,** 649-661 (2012).

4. van der Windt, G. J. & Pearce E. L. Metabolic switching and fuel choice during T-cell differentiation and memory development. *Immunol. Rev.* **249,** 27-42 (2012).

5. Scharping, N. E. et al. The tumor microenvironment represses T cell mitochondrial biogenesis to drive intratumoral T cell metabolic insufficiency and dysfunction. *Immunity* **45,** 374-388 (2016).

6. Frauwirth, K. A. et al. The CD28 signaling pathway regulates glucose metabolism. *Immunity* **16,** 769-777 (2002).

7. Zhou, Y., Wu, H., Zhao, M., Chang, C. & Lu, Q. The Bach family of transcription factors: a comprehensive review. *Clin. Rev. Allergy Immunol.* **50,** 345-356 (2016).

8. Ochiai, K., Muto, A., Tanaka, H., Takahashi, S. & Igarashi, K. Regulation of the plasma cell transcription factor Blimp-1 gene by Bach2 and Bcl6. *Int. Immunol.* **20,** 453-460 (2008).

9. Ise, W. et al. The transcription factor BATF controls the global regulators of class-switch recombination in both B cells and T cells. *Nat. Immunol.* **12,** 536-543 (2011).

10. Xin, G. et al. A critical role of IL-21-induced BATF in sustaining CD8-T-cell-mediated chronic viral control. *Cell Rep.* **13,** 1118-1124 (2015).

11. Kurachi, M. et al. The transcription factor BATF operates as an essential differentiation checkpoint in early effector CD8^+^ T cells. *Nat. Immunol.* **15,** 373-383 (2014).

12. Yoshida, K. et al. Bcl6 controls granzyme B expression in effector CD8^+^ T cells. *Eur. J. Immunol.* **36,** 3146-3156 (2006).

13. Leong, Y. A. et al. CXCR5^+^ follicular cytotoxic T cells control viral infection in B cell follicles. *Nat. Immunol.* **17,** 1187-1196 (2016).

14. Vasanwala, F. H., Kusam, S., Toney, L. M. & Dent A. L. Repression of AP-1 function: a mechanism for the regulation of Blimp-1 expression and B lymphocyte differentiation by the B cell lymphoma-6 protooncogene. *J. Immunol.* **169,** 1922-1929 (2002).

15. Chen, L. & Flies, D. B. Molecular mechanisms of T cell co-stimulation and co-inhibition. *Nat. Rev. Immunol.* **13,** 227-242 (2013).

16. Macian, F. NFAT proteins: key regulators of T-cell development and function. *Nat. Rev. Immunol.* **5,** 472-484 (2005).

17. Viganὸ, S. et al. CD160-associated CD8 T-cell functional impairment is independent of PD-1 expression. *PLoS Pathog.* **10,** e1004380 (2014).

18. Maghazachi, A. A. Insights into seven and single transmembrane-spanning domain receptors and their signaling pathways in human natural killer cells. *Pharmacol. Rev.* **57,** 339-357 (2005).

19. Kwon, H. J. et al. Stepwise phosphorylation of p65 promotes NF-kB activation and NK cell responses during target cell recognition. *Nat. Commun.* **7,** 11686 (2016).

20. Genot, E. M. et al. The T-cell receptor regulates Akt (protein kinase B) via a pathway involving Rac1 and phosphatidylinositide 3-kinase. *Mol. Cell Biol.* **20,** 5469-5478 (2000).

21. Gamper, C. J., Agoston, A. T., Nelson, W. G. & Powell, J. D. Identification of DNA methyltransferase 3a as a T cell receptor-induced regulator of Th1 and Th2 differentiation. *J. Immunol.* **183,** 2267-2276 (2009).

22. Rincόn, M. & Flavell R. A. AP-1 transcriptional activity requires both T-cell receptor-mediated and co-stimulatory signals in primary T lymphocytes. *EMBO J*. **13,** 4370-4381 (1994).

23. Thaker Y. R., Schneider, H. & Rudd, C. E. TCR and CD28 activate the transcription factor NF-κB in T-cells via distinct adaptor signaling complexes. *Immunol. Lett.* **163,** 113-119 (2015).

24. Müller, M. R. & Rao A. NFAT, immunity and cancer: a transcription factor comes of age. *Nat. Rev. Immunol.* **10,** 645-656 (2010).

25. Xia, Y., Jeffrey Medeiros, L. & Young, K.H. Signaling pathway and dysregulation of PD1 and its ligands in lymphoid malignancies. *Biochim. Biophys. Acta.* **1865,** 58-71 (2016).

26. Pollizzi, K. N. & Powell, J. D. Integrating canonical and metabolic signalling programmes in the regulation of T cell responses. *Nat. Rev. Immunol.* **14,** 435-446 (2014).

27. Dustin, M. L. PKC-θ: hitting the bull’s eye. *Nat. Immunol.* **12,** 1031-1032 (2011).

28. Wang, Y., et al. A conserved CXXC motif in CD3epsilon is critical for T cell development and TCR signaling. *PLoS Biol.* **7,** e1000253 (2009).

29. Youngblood, B. et al. Chronic virus infection enforces demethylation of the locus that encodes PD-1 in antigen-specific CD8^+^ T cells. *Immunity* **35,** 400-412 (2011).

30. Ladle, B. H. et al. De novo DNA methylation by DNA methyltransferase 3a controls early effector CD8^+^ T-cell fate decisions following activation. *Proc. Natl. Acad. Sci. U S A* **113,** 10631-10636 (2016).

31. Scharer , C. D., [Barwick, B. G](https://www.ncbi.nlm.nih.gov/pubmed/?term=Barwick%20BG%5BAuthor%5D&cauthor=true&cauthor_uid=23956425).,Y[oungblood, B. A](https://www.ncbi.nlm.nih.gov/pubmed/?term=Youngblood%20BA%5BAuthor%5D&cauthor=true&cauthor_uid=23956425)., [Ahmed, R](https://www.ncbi.nlm.nih.gov/pubmed/?term=Ahmed%20R%5BAuthor%5D&cauthor=true&cauthor_uid=23956425). & [Boss, J. M](https://www.ncbi.nlm.nih.gov/pubmed/?term=Boss%20JM%5BAuthor%5D&cauthor=true&cauthor_uid=23956425). Global DNA methylation remodeling accompanies CD8 T cell effector function. *J. Immunol.* **191,** 3419-3429 (2013)

32. Bain, G. et al. Regulation of the helix-loop-helix proteins, E2A and Id3, by the Ras-ERK MAPK cascade. *Nat. Immunol.* **2,** 165-171 (2001).

33. Skerka, C., Decker, E. L. & Zipfel P.F. A regulatory element in the human interleukin 2 gene promoter is a binding site for the zinc finger proteins Sp1 and EGR-1. *J. Biol. Chem.* **270,** 22500-22506 (1995).

34. Lin, J. X. & Leonard W. J. The immediate-early gene product Egr-1 regulates the human interleukin-2 receptor beta-chain promoter through noncanonical Egr and Sp1 binding sites. *Mol*. *Cell Biol.* **17,** 3714-3722 (1997).

35. Miao, T. et al. Egr2 and 3 control adaptive immune responses by temporally uncoupling expansion from T cell differentiation. *J. Exp. Med.* **214,** 1787-1808 (2017).

36. Martinez, G. J. et al. The transcription factor NFAT promotes exhaustion of activated CD8^+^ T cells. *Immunity* **42,** 265-278 (2015).

37. Singer, M. et al. A distinct gene module for dysfunction uncoupled from activation in tumor-infiltrating T cells. *Cell* **166,** 1500-1511.e9 (2016).

38. Viré, E. et al. The Polycomb group protein EZH2 directly controls DNA methylation. *Nature* **439,** 871-874 (2006).

39. Lee, D. U., Avni, O., Chen, L. & Rao A. A distal enhancer in the interferon-gamma (IFN-gamma) locus revealed by genome sequence comparison. *J. Biol. Chem.* **279,** 4802-4810 (2004).

40. Martins, G. & Calame, K. Regulation and functions of Blimp-1 in T and B lymphocytes. *Annu. Rev. Immunol.* **26,** 133-169 (2008).

41. Liao, W., Lin, J. X. & Leonard, W. J. Interleukin-2 at the crossroads of effector responses, tolerance, and immunotherapy. *Immunity* **38,** 13-25 (2013).

42. Bally, A. P., Austin, J. W. & Boss, J. M. Genetic and epigenetic regulation of PD-1 expression. *J. Immunol.* **196,** 2431-2437 (2016).

43. Oestreich, K. J., Mohn, S. E. & Weinmann, A.S. Molecular mechanisms that control the expression and activity of Bcl-6 in TH1 cells to regulate flexibility with a TFH-like gene profile. *Nat. Immunol.* **13,** 405-411 (2012).

44. Gray, S. M., Amezquita, R. A., Guan, T., Kleinstein, S. H. & Kaech, S. M. Polycomb repressive complex 2-mediated chromatin repression guides effector CD8^+^ T cell terminal differentiation and loss of multipotency. *Immunity* **46,** 596-608 (2017).

45. Staron, M. M. et al. The transcription factor FoxO1 sustains expression of the inhibitory receptor PD-1 and survival of antiviral CD8^+^ T cells during chronic infection. *Immunity* **41,** 802-814 (2014).

46. Pauken, K. E. & Wherry, E. J. Overcoming T cell exhaustion in infection and cancer. *Trends Immunol.* **36,** 265-276 (2015).

47. Wang, Y. et al. GATA-3 controls the maintenance and proliferation of T cells downstream of TCR and cytokine signaling. *Nat. Immunol.* **14,** 714-722 (2013).

48. Hough, K. P., Chisolm, D. A. & Weinmann, A. S. Transcriptional regulation of T cell metabolism. *Mol. Immunol.* **68,** 520-526 (2015).

49. Chen, G., Hitomi, M., Han, J. & Stacey D. W. The p38 pathway provides negative feedback for Ras proliferative signaling. *J. Biol. Chem.* **275,** 38973-38980 (2000).

50. Nakayamada, S. et al. Type I IFN induces binding of STAT1 to Bcl6: divergent roles of STAT family transcription factors in the T follicular helper cell genetic program. *J. Immunol.* **192,** 2156-2166 (2014).

51. Zhou, G & Ono, S. J. Induction of BCL-6 gene expression by interferon-gamma and identification of an IRE in exon I. *Exp. Mol. Pathol.* **78,** 25-35 (2005).

52. Friedman, R. L., Manly, S. P., McMahon, M., Kerr, I. M. & Stark, G. R. Transcriptional and posttranscriptional regulation of interferon-induced gene expression in human cells. *Cell* **38,** 745-755 (1984).

53. Platanias, L. C. Mechanisms of type-1 and type-II-interferon-mediated signalling. *Nat. Rev. Immunol*. **5,** 375-386 (2005).

54. Au-Yeung, N., Mandhana, R. & Horvath, C. M. Transcriptional regulation by STAT1 and STAT2 in the interferon JAK-STAT pathway. *JAKSTAT* **2,** e23931 (2013).

55. Ramos, H. J., Davis, A. M., George, T. C. & Farra, J. D. IFN-alpha is not sufficient to drive Th1 development due to lack of stable T-bet expression. *J. Immunol.* **179,** 3792-3803 (2007).

56. Schurich, A. et al. Distinct metabolic requirements of exhausted and functional virus-specific CD8 T cells in the same host. *Cell Rep.* **16,** 1243-1252 (2016).

57. Radaeva, S. et al. Interferon-gamma inhibits interferon-alpha signalling in hepatic cells: evidence for the involvement of STAT1 induction and hyperexpression of STAT1 in chronic hepatitis C. *Biochem. J.* **379,** 199-208 (2004).

58. Kuroda, S. et al. Basic leucine zipper transcription factor, ATF-like (BATF) regulates epigenetically and energetically effector CD8 T-cell differentiation via Sirt1 expression. *Proc. Natl. Acad. Sci. U S A* **108,** 14885-14889 (2011).

59. Xin, A. et al. A molecular threshold for effector CD8^+^ T cell differentiation controlled by transcription factors Blimp-1 and T-bet. *Nat. Immunol.* **17,** 422-432 (2016).

60. Bacon, C. M. et al. Interleukin 12 induces tyrosine phosphorylation and activation of STAT4 in human lymphocytes. *Proc Natl Acad Sci U S A* **92,** 7307-7311 (1995).

61. Stark, R., Hartung, A., Zehn, D., Frentsch, M. & Thiel, A. IL-12-mediated STAT4 signaling and TCR signal strength cooperate in the induction of CD40L in human and mouse CD8^+^ T cells. *Eur. J. Immunol.* **43,** 1511-1517 (2013).

62. Joshi, N. S. et al. Inflammation directs memory precursor and short-lived effector CD8^+^ T cell fates via the graded expression of T-bet transcription factor. *Immunity* **27,** 281-295 (2007).

63. Wan, C. K. et al. Opposing roles of STAT1 and STAT3 in IL-21 function in CD4^+^ T cells. *Proc Natl Acad Sci U S A* **112,** 9394-9399 (2015).

64. Rao, R. R., Li, Q., Gubbels Bupp, M. R. & Shrikant, P. A. Transcription factor Foxo1 represses T-bet-mediated effector functions and promotes memory CD8^+^ T cell differentiation. *Immunity* **36**, 374-387 (2012).

65. Oestreich, K. J. et al. Bcl-6 directly represses the gene program of the glycolysis pathway. *Nat. Immunol.* **15,** 957-964 (2014).

66. Ouyang, W., Beckett, O., Flavell, R. A. & Li, M. O. An essential role of the Forkhead-box transcription factor Foxo1 in control of T cell homeostasis and tolerance. *Immunity* **30,** 358-371 (2009).

67. Wan, Y. Y. GATA3: a master of many trades in immune regulation. *Trends Immunol.* **35,** 233-242 (2014).

68. Beltra, J. C. et al. IL2Rß-dependent signals drive terminal exhaustion and suppress memory development during chronic viral infection. *Proc. Natl. Acad. Sci. U S A* **113,** E5444-5453 (2016).

69. Raczkowski, F. et al. The transcription factor Interferon Regulatory Factor 4 is required for the generation of protective effector CD8^+^ T cells. *Proc. Natl. Acad. Sci. U S A* **110,** 15019-15024 (2013).

70. De Santa, F. et al. The histone H3 lysine-27 demethylase Jmjd3 links inflammation to inhibition of polycomb-mediated gene silencing. *Cell* **130,** 1083-1094 (2007).

71. Xu, L. et al. The transcription factor TCF-1 initiates the differentiation of T_FH_ cells during acute viral infection. *Nat. Immunol.* **16,** 991-999 (2015).

72. Choi, Y. S. et al. LEF-1 and TCF-1 orchestrate T_FH_ differentiation by regulating differentiation circuits upstream of the transcriptional repressor Bcl6. *Nat. Immunol.* **16,** 980-990 (2015).

73. Zhou, X. et al. Differentiation and persistence of memory CD8^+^ T cells depend on T cell factor 1. *Immunity* **33,** 229-240 (2010).

74. Yu, Q. et al. T cell factor 1 initiates the T helper type 2 fate by inducing the transcription factor GATA-3 and repressing interferon-gamma. *Nat. Immunol.* **10,** 992-999 (2009).

75. Xing, S. et al. Tcf1 and Lef1 transcription factors establish CD8^+^ T cell identity through intrinsic HDAC activity. *Nat. Immunol.* **17,** 695-703 (2016).

76. Collins, S. et al. Opposing regulation of T cell function by Egr-1/NAB2 and Egr-2/Egr-3. *Eur. J. Immunol.* **38,** 528-536 (2008).

77. Lazarczyk, M. et al. EVER proteins, key elements of the natural anti-human papillomavirus barrier, are regulated upon T-cell activation. *PLoS One* **7,** e39995 (2012).

78. Rosner, M., [Siegel, N](https://www.ncbi.nlm.nih.gov/pubmed/?term=Siegel%20N%5BAuthor%5D&cauthor=true&cauthor_uid=19145465)., [Valli, A](https://www.ncbi.nlm.nih.gov/pubmed/?term=Valli%20A%5BAuthor%5D&cauthor=true&cauthor_uid=19145465)., [Fuchs, C](https://www.ncbi.nlm.nih.gov/pubmed/?term=Fuchs%20C%5BAuthor%5D&cauthor=true&cauthor_uid=19145465)., & [Hengstschläger M](https://www.ncbi.nlm.nih.gov/pubmed/?term=Hengstschl%C3%A4ger%20M%5BAuthor%5D&cauthor=true&cauthor_uid=19145465). mTOR phosphorylated at S2448 binds to raptor and rictor. *Amino Acids* **38,** 223-228 (2010).

79. Kiani, A. et al. Regulation of interferon-gamma gene expression by nuclear factor of activated T cells. *Blood* **98,** 1480-1488 (2001).

80. Serfling, E., Chuvpilo, S., Liu, J., Höfer, T. & Palmetshofer, A. NFATc1 autoregulation: a crucial step for cell-fate determination. *Trends Immunol.* **27,** 461-469 (2006).

81. [Martínez-González, J](https://www.ncbi.nlm.nih.gov/pubmed/?term=Mart%C3%ADnez-Gonz%C3%A1lez%20J%5BAuthor%5D&cauthor=true&cauthor_uid=15664387). & Badimon, L. The NR4A subfamily of nuclear receptors: new early genes regulated by growth factors in vascular cells. *Cardiovasc. Res.* **65,** 609-618 (2005).

82. Zhou, B. et al. Regulation of the murine Nfatc1 gene by NFATc2. *J. Biol. Chem.* **277,** 10704-10711 (2002).

83. Shaffer, A. L., [Emre, N. C](https://www.ncbi.nlm.nih.gov/pubmed/?term=Emre%20NC%5BAuthor%5D&cauthor=true&cauthor_uid=19383829)., [Romesser, P. B](https://www.ncbi.nlm.nih.gov/pubmed/?term=Romesser%20PB%5BAuthor%5D&cauthor=true&cauthor_uid=19383829). & [Staudt, L. M](https://www.ncbi.nlm.nih.gov/pubmed/?term=Staudt%20LM%5BAuthor%5D&cauthor=true&cauthor_uid=19383829). IRF4: Immunity. Malignancy! Therapy? *Clin. Cancer Res.* **15,** 2954-2961 (2009).

84. [Fassett, M. S](https://www.ncbi.nlm.nih.gov/pubmed/?term=Fassett%20MS%5BAuthor%5D&cauthor=true&cauthor_uid=22345564)., [Jiang, W](https://www.ncbi.nlm.nih.gov/pubmed/?term=Jiang%20W%5BAuthor%5D&cauthor=true&cauthor_uid=22345564)., [D'Alise, A. M](https://www.ncbi.nlm.nih.gov/pubmed/?term=D%27Alise%20AM%5BAuthor%5D&cauthor=true&cauthor_uid=22345564)., [Mathis, D](https://www.ncbi.nlm.nih.gov/pubmed/?term=Mathis%20D%5BAuthor%5D&cauthor=true&cauthor_uid=22345564). & [Benoist C](https://www.ncbi.nlm.nih.gov/pubmed/?term=Benoist%20C%5BAuthor%5D&cauthor=true&cauthor_uid=22345564). Nuclear receptor Nr4a1 modulates both regulatory T-cell (Treg) differentiation and clonal deletion. *Proc. Natl. Acad. Sci. U S A* **109,** 3891-3896 (2012).

85. Nowyhed, H. N., Huynh, T. R., [Thomas, G. D](https://www.ncbi.nlm.nih.gov/pubmed/?term=Thomas%20GD%5BAuthor%5D&cauthor=true&cauthor_uid=26363057)., Blatchley A. & [Hedrick, C. C](https://www.ncbi.nlm.nih.gov/pubmed/?term=Hedrick%20CC%5BAuthor%5D&cauthor=true&cauthor_uid=26363057). Cutting edge: the orphan nuclear receptor Nr4a1 regulates CD8^+^ T cell expansion and effector function through direct repression of Irf4. *J. Immunol*. **195,** 3515-3519 (2015).

86. Mognol, G. P. et al. Exhaustion-associated regulatory regions in CD8^+^ tumor-infiltrating T cells. *Proc. Natl. Acad. Sci. U S A* **114,** E2776-E2785 (2017).

87. Odagiu, L., Boulet, S., Daudelin, J. F. & Labrecque, N. NR4A3 controls CD8^+^ T cell metabolism and differentiation. *J. Immunol.* **196** (1 Suppl), 57.8 (2016).

88. Bengsch, B. et al. Bioenergetic insufficiencies due to metabolic alterations regulated by the inhibitory receptor PD-1 are an early driver of CD8^+^ T cell exhaustion. *Immunity* **45,** 358-373 (2016).

89. Kamphorst, A. O. et al. Rescue of exhausted CD8 T cells by PD-1-targeted therapies is CD28-dependent. *Science* **355,** 1423-1427 (2017).

90. Quigley, M. et al. Transcriptional analysis of HIV-specific CD8^+^ T cells shows that PD-1 inhibits T cell function by upregulating BATF. *Nat. Med.* **16,** 1147-1151 (2010).

91. Boussiotis, V.A. Molecular and biochemical aspects of the PD-1 checkpoint pathway. *N. Eng. J. Med.* **375,** 1767-1778 (2016).

92. Shin, H. M. et al. Epigenetic modifications induced by Blimp-1 regulate CD8^+^ T cell memory progression during acute virus infection. *Immunity* **39,** 661-675 (2013).

93. Cimmino, L. et al. Blimp-1 attenuates Th1 differentiation by repression of ifng, tbx21, and bcl6 gene expression. *J. Immunol.* **181,** 2338-2347 (2008).

94. Gong, D. & Malek, T. R. Cytokine-dependent Blimp-1 expression in activated T cells inhibits IL-2 production. *J. Immunol.* **178,** 242-252 (2007).

95. Lin, Y., Wong, K. & Calame, K. Repression of c-myc transcription by BLIMP-1, an inducer of terminal B cell differentiation. *Science* **276,** 596-599 (1997).

96. Lu, P. et al. Blimp-1 represses CD8 T cell expression of PD-1 using a feed-forward transcriptional circuit during acute viral infection. *J. Exp. Med.* **211,** 515-527 (2014).

97. Lin, X., [O'Mahony, A](https://www.ncbi.nlm.nih.gov/pubmed/?term=O%27Mahony%20A%5BAuthor%5D&cauthor=true&cauthor_uid=10733597)., [Mu, Y](https://www.ncbi.nlm.nih.gov/pubmed/?term=Mu%20Y%5BAuthor%5D&cauthor=true&cauthor_uid=10733597)., [Geleziunas, R](https://www.ncbi.nlm.nih.gov/pubmed/?term=Geleziunas%20R%5BAuthor%5D&cauthor=true&cauthor_uid=10733597). & [Greene, W. C](https://www.ncbi.nlm.nih.gov/pubmed/?term=Greene%20WC%5BAuthor%5D&cauthor=true&cauthor_uid=10733597). Protein kinase C-theta participates in NF-kappaB activation induced by CD3-CD28 costimulation through selective activation of IkappaB kinase beta. *Mol. Cell Biol.* **20,** 2933-2940 (2000).

98. Pham, D., [Vincentz, J. W](https://www.ncbi.nlm.nih.gov/pubmed/?term=Vincentz%20JW%5BAuthor%5D&cauthor=true&cauthor_uid=22685315)., [Firulli, A. B](https://www.ncbi.nlm.nih.gov/pubmed/?term=Firulli%20AB%5BAuthor%5D&cauthor=true&cauthor_uid=22685315). & [Kaplan, M. H](https://www.ncbi.nlm.nih.gov/pubmed/?term=Kaplan%20MH%5BAuthor%5D&cauthor=true&cauthor_uid=22685315). Twist1 regulates Ifng expression in Th1 cells by interfering with Runx3 function. *J. Immunol.* **189,** 832-840 (2012).

99. Jeong, J. & Eide, D. J. The SLC39 family of zinc transporters. *Mol. Aspects Med.* **34,** 612-619 (2013).

100. Oh, H. M. et al. STAT3 protein promotes T-cell survival and inhibits interleukin-2 production through up-regulation of Class O Forkhead transcription factors. *J. Biol. Chem.* **286,** 30888-30897 (2011).

101. Gil, M. P. et al. Regulating type 1 IFN effects in CD8 T cells during viral infections: changing STAT4 and STAT1 expression for function. *Blood* **120,** 3718-3728 (2012).

102. Pepper, M., [Pagán, A. J](https://www.ncbi.nlm.nih.gov/pubmed/?term=Pag%C3%A1n%20AJ%5BAuthor%5D&cauthor=true&cauthor_uid=22018468)., [Igyártó, B. Z](https://www.ncbi.nlm.nih.gov/pubmed/?term=Igy%C3%A1rt%C3%B3%20BZ%5BAuthor%5D&cauthor=true&cauthor_uid=22018468)., [Taylor, J. J](https://www.ncbi.nlm.nih.gov/pubmed/?term=Taylor%20JJ%5BAuthor%5D&cauthor=true&cauthor_uid=22018468). & [Jenkins, M. K](https://www.ncbi.nlm.nih.gov/pubmed/?term=Jenkins%20MK%5BAuthor%5D&cauthor=true&cauthor_uid=22018468). Opposing signals from the Bcl6 transcription factor and the interleukin-2 receptor generate T helper 1 central and effector memory cells. *Immunity* **35,** 583-595 (2011).

103. Kao, C. et al. Transcription factor T-bet represses expression of the inhibitory receptor PD-1 and sustains virus-specific CD8^+^ T cell responses during chronic infection. *Nat. Immunol.* **12,** 663-671 (2011).

104. Anderson, A. C. et al. T-bet, a Th1 transcription factor regulates the expression of Tim-3. *Eur. J. Immunol.* **40,** 859-866 (2010).

105. Lazarevic, V.  [Glimcher, L. H](https://www.ncbi.nlm.nih.gov/pubmed/?term=Glimcher%20LH%5BAuthor%5D&cauthor=true&cauthor_uid=24113868). & [Lord, G. M](https://www.ncbi.nlm.nih.gov/pubmed/?term=Lord%20GM%5BAuthor%5D&cauthor=true&cauthor_uid=24113868). T-bet: a bridge between innate and adaptive immunity. *Nat. Rev. Immunol.* **13,** 777-789 (2013).

106. Omilusik, K. D. et al. Transcriptional repressor ZEB2 promotes terminal differentiation of CD8^+^ effector and memory T cell populations during infection. *J. Exp. Med.* **212,** 2027-2039 (2015).

107. Tsagaratou, A., [Lio, C. J](https://www.ncbi.nlm.nih.gov/pubmed/?term=Lio%20CJ%5BAuthor%5D&cauthor=true&cauthor_uid=28408905)., [Yue, X](https://www.ncbi.nlm.nih.gov/pubmed/?term=Yue%20X%5BAuthor%5D&cauthor=true&cauthor_uid=28408905). & [Rao, A](https://www.ncbi.nlm.nih.gov/pubmed/?term=Rao%20A%5BAuthor%5D&cauthor=true&cauthor_uid=28408905). TET methylcytosine oxidases in T cell and B cell development and function. *Front Immunol.* **8,** 220 (2017)

108. Tsagaratou, A. et al. TET proteins regulate the lineage specification and TCR-mediated expansion of iNKT cells. *Nat. Immunol.* **18,** 45-53 (2017).

109. McPherson, R. C. et al. Epigenetic modification of the PD-1 (Pdcd1) promoter in effector CD4^+^ T cells tolerized by peptide immunotherapy. *Elife* **3**, e03416 (2014).

110. Martinet, L. & Smyth, M. J. Balancing natural killer cell activation through paired receptors. *Nat. Rev. Immunol*. **15,** 243-254 (2015).

111. Beyer, M. et al. Tumor-necrosis factor impairs CD4^+^ T cell-mediated immunological control in chronic viral infection. *Nat. Immunol.* **17,** 593-603 (2016).

112. Nam, K. O. et al. Cross-linking of 4-1BB activates TCR-signaling pathways in CD8^+^ T lymphocytes. *J. Immunol.* **174,** 1898-1905 (2005).

113. Lee, D. Y. et al. 4-1BB signaling activates the T cell factor 1 effector/β-catenin pathway with delayed kinetics via ERK signaling and delayed PI3K/AKT activation to promote the proliferation of CD8^+^ T cells. *PLoS One* **8,** e69677 (2013).

114. Katsuyama, E. et al. Downregulation of miR-200a-3p, targeting CtBP2 complex, is involved in the hypoproduction of IL-2 in systemic lupus erythematosus-derived T cells. *J. Immunol.* **198,** 4268-4276 (2017).

115. Dominguez, C. X. et al. The transcription factors ZEB2 and T-bet cooperate to program cytotoxic T cell terminal differentiation in response to LCMV viral infection. *J. Exp. Med.* **212,** 2041-2056 (2015).

116. Hernandez-Chacon, J. A. et al. Costimulation through the CD137/4-1BB pathway protects human melanoma tumor-infiltrating lymphocytes from activation-induced cell death and enhances antitumor effector function. *J. Immunother.* **34,** 236-250 (2011).

117. Lee, H. W. et al. 4-1BB promotes the survival of CD8^+^ T lymphocytes by increasing expression of Bcl-xL and Bfl-1. *J. Immunol.* **169,** 4882-4888 (2002).

118. Gallo, E. M., Canté-Barrett, K. & Crabtree, G. R. Lymphocyte calcium signaling from membrane to nucleus. *Nat. Immunol.* **7,** 25-32 (2006).

119. Chuvpilo, S. et al. Alternative polyadenylation events contribute to the induction of NF-ATc in effector T cells. *Immunity* **10,** 261-269 (1999).

120. Diehn, M. et al. Genomic expression programs and the integration of the CD28 costimulatory signal in T cell activation. *Proc. Natl. Acad. Sci. U S A* **99,** 11796-11801 (2002).

121. Araki, K., Youngblood, B. & Ahmed, R. The role of mTOR in memory CD8 T-cell differentiation. *Immunol. Rev.* **235,** 234-243 (2010).

122. Keating, R. & McGargill, M. A. mTOR regulation of lymphoid cells in immunity to pathogens. *Front. Immunol.* **7,** 180 (2016).

123. Schulze-Luehrmann, J. & Ghosh, S. Antigen-receptor signaling to nuclear factor kappa B. *Immunity* **25,** 701-715 (2006).

124. Gorentla, B. K. & Zhong, X. P. T cell receptor signal transduction in T lymphocytes. *J. Clin. Cell. Immunol.* **2012,** 5 (2012).

125. Cunningham, N. R. et al. Immature CD4^+^CD8^+^ thymocytes and mature T cells regulate Nur77 distinctly in response to TCR stimulation. *J. Immunol.* **177,** 6660-6666 (2006).

126. Han, Y. H. et al. Regulation of Nur77 nuclear export by c-Jun N-terminal kinase and Akt. *Oncogene* **25,** 2974-2986 (2006).

127. Tullai, J. W., Tacheva, S., Owens, L. J., Graham, J. R. & Cooper, G. M. AP-1 is a component of the transcriptional network regulated by GSK-3 in quiescent cells. *PLoS One* **6,** e20150 (2011).

128. Kang, H. J. et al. Retinoic acid and its receptors repress the expression and transactivation functions of Nur77: a possible mechanism for the inhibition of apoptosis by retinoic acid. *Exp. Cell Res.* **256,** 545-554 (2000).

129. Glasmacher, E. et al. A genomic regulatory element that directs assembly and function of immune-specific AP-1-IRF complexes. *Science* **338,** 975-980 (2012).

130. Li, P. et al. BATF-JUN is critical for IRF4-mediated transcription in T cells. *Nature* **490,** 543-546 (2012).

131. Deppmann, C. D., Thornton, T. M., Utama, F. E. & Taparowsky, E. J. Phosphorylation of BATF regulates DNA binding: a novel mechanism for AP-1 (activator protein-1) regulation. *Biochem. J.* **374,** 423-431 (2003).

132. Murphy, T. L., Tussiwand, R. & Murphy, K. M. Specificity through cooperation: BATF-IRF interactions control immune-regulatory networks. *Nat. Rev. Immunol.* **13,** 499-509 (2013).

133. Tsukumo, S. et al. Bach2 maintains T cells in a naive state by suppressing effector memory-related genes. *Proc. Natl. Acad. Sci. U S A* **110,** 10735-10740 (2013).

134. Hu, G. & Chen, J. A genome-wide regulatory network identifies key transcription factors for memory CD8^+^ T-cell development. *Nat. Commun.* **4,** 2830 (2013).

135. Man, K. et al. Transcription factor IRF4 promotes CD8^+^ T cell exhaustion and limits the development of memory-like T cells during chronic infection. *Immunity* **47,** 1129-1141.e5 (2017).

136. Ciofani, M. et al. A validated regulatory network for Th17 cell specification. *Cell* **151,** 289-303 (2012).

137. Crotty, S., Johnston, R. J. & Schoenberger, S. P. Effectors and memories: Bcl-6 and Blimp-1 in T and B lymphocyte differentiation. *Nat. Immunol.* **11,** 114-120 (2010).

138. Zhu, L. et al. Blimp-1 impairs T cell function via upregulation of TIGIT and PD-1 in patients with acute myeloid leukemia. *J. Hematol. Oncol.* **10,** 124 (2017).

139. Shin, H. et al. A role for the transcriptional repressor Blimp-1 in CD8^+^ T cell exhaustion during chronic viral infection. *Immunity* **31,** 309-320 (2009).

140. Welsh, R. M. Blimp hovers over T cell immunity. *Immunity* **31,** 178-180 (2009).

141. Rutishauser, R. L. et al. Transcriptional repressor Blimp-1 promotes CD8^+^ T cell terminal differentiation and represses the acquisition of central memory T cell properties. *Immunity* **31,** 296-308 (2009).

142. Ji, Y. et al. Repression of the DNA-binding inhibitor Id3 by Blimp-1 limits the formation of memory CD8^+^ T cells. *Nat. Immunol.* **12,** 1230-1237 (2011).

143. Wu, T. H., Zhen, Y., Zeng, C., Yi, H. F. & Zhao, Y. B and T lymphocyte attenuator interacts with CD3zeta and inhibits tyrosine phosphorylation of TCRzeta complex during T-cell activation. *Immunol. Cell Biol.* **85,** 590-595 (2007).

144. Sharma, K. et al. Death the Fas way: regulation and pathophysiology of CD95 and its ligand. *Pharmacol. Ther.* **88,** 333-347 (2000).

145. Dhein, J., Walczak, H., Baumler, C., Debatin, K. M. & Krammer, P. H. Autocrine T-cell suicide mediated by APO-1/(Fas/CD95). *Nature* **373,** 438-441 (1995).

146. Rubio, I. et al. TCR-induced activation of Ras proceeds at the plasma membrane and requires palmitoylation of N-Ras. *J. Immunol.* **185,** 3536-3543 (2010).

147. He, S. et al. Ezh2 phosphorylation state determines its capacity to maintain CD8^+^ T memory precursors for antitumor immunity. *Nat. Commun.* **8,** 2125 (2017).

148. Dobenecker, M. W. et al*.* Signaling function of PRC2 is essential for TCR-driven T cell responses. *J. Exp. Med.* **215,** 1101-1113 (2018).

149. Buchbinder, E. I. & Desai, A. CTLA-4 and PD-1 pathways: similarities, differences, and implications of their inhibition. *Am. J. Clin. Oncol.* **39,** 98-106 (2016).

150. He, R. et al. Follicular CXCR5- expressing CD8^+^ T cells curtail chronic viral infection. *Nature* **537,** 412-428 (2016).

151. Singh, R. et al. Egr2 and 3 inhibit T-bet-mediated IFN- γ production in T cells. *J. Immunol.* **198,** 4394-4402 (2017).

152. Zheng, Y. et al. Egr2-dependent gene expression profiling and ChIP-Seq reveal novel biologic targets in T cell anergy. *Mol. Immunol.* **55,** 283-291 (2013).

153. Mittelstadt, P. R. & Ashwell, J. D. Cyclosporin A-sensitive transcription factor Egr-3 regulates Fas ligand expression. *Mol. Cell. Biol.* **18,** 3744-3751 (1998).

154. Kavurma, M. M. & Khachigian, L. M. Signaling and transcriptional control of Fas ligand gene expression. *Cell Death Differ.* **10,** 36-44 (2003).

155. Cruz-Guilloty, F. et al. Runx3 and T-box proteins cooperate to establish the transcriptional program of effector CTLs. *J. Exp. Med.* **206,** 51-59 (2009).

156. Zhang, J. et al. Regulation of fas ligand expression during activation-induced cell death in T cells by p38 mitogen-activated protein kinase and c-Jun NH2-terminal kinase. *J. Exp. Med.* **191,** 1017-1030 (2000).

157. Daitoku, H., Yamagata, K., Matsuzaki, H., Hatta, M. & Fukamizu, A. Regulation of PGC-1 promoter activity by protein kinase B and the forkhead transcription factor FKHR. *Diabetes* **52,** 642-649 (2003).

158. Mu, X. et al. IGF-II-mediated downregulation of peroxisome proliferator-activated receptor-γ coactivator-1α in myoblast cells involves PI3K/Akt/FoxO1 signaling pathway. *Mol. Cell. Biochem.* **432,** 199-208 (2017).

159. Zhang, L. et al. Mammalian target of rapamycin complex 2 controls CD8 T cell memory differentiation in a Foxo1-dependent manner. *Cell Rep.* **14,** 1206-1217 (2016).

160. McKinney, E. F. & Smith, K. G. C. Metabolic exhaustion in infection, cancer and autoimmunity. *Nat. Immunol.* **19,** 213-221 (2018).

161. Hess Michelini, R., Doedens, A. L., Goldrath, A. W. & Hedrick, S. M. Differentiation of CD8 memory T cells depends on Foxo1. *J. Exp. Med.* **210,** 1189-1200 (2013).

162. Narravula, S. & Colgan, S. P. Hypoxia-inducible factor 1-mediated inhibition of peroxisome proliferator-activated receptor alpha expression during hypoxia. *J. Immunol.* **166,** 7543-7548 (2001).

163. Tan, Z. et al. The role of PGC1α in cancer metabolism and its therapeutic implications. *Mol. Cancer Ther.* **15,** 774-782 (2016).

164. Menner, A. J. et al. Id3 controls cell death of 2B4^+^ virus-specific CD8^+^ T cells in chronic viral infection. *J. Immunol.* **195,** 2103-2114 (2015).

165. Yang, C. Y. et al. The transcriptional regulators Id2 and Id3 control the formation of distinct memory CD8^+^ T cell subsets. *Nat. Immunol.* **12,** 1221-1229 (2011).

166. Miyazaki, M. et al. The opposing roles of the transcription factor E2A and its antagonist Id3 that orchestrate and enforce the naive fate of T cells. *Nat. Immunol.* **12,** 992-1001 (2011).

167. Kirchhoff, S. et al. Viral IFN-regulatory factors inhibit activation-induced cell death via two positive regulatory IFN-regulatory factor 1-dependent domains in the CD95 ligand promoter. *J. Immunol.* **168,** 1226-1234 (2002).

168. Takemoto, N., Intlekofer, A. M., Northrup, J. T., Wherry, E. J. & Reiner, S. L. Cutting edge: IL-12 inversely regulates T-bet and eomesodermin expression during pathogen-induced CD8^+^ T cell differentiation. *J. Immunol.* **177,** 7515-7519 (2006).

169. Ylikoski, E. et al. IL-12 up-regulates T-bet independently of IFN-gamma in human CD4^+^ T cells. *Eur. J. Immunol.* **35,** 3297-3306 (2005).

170. Chew, G. M. et al. TIGIT marks exhausted T cells, correlates with disease progression, and serves as a target for immune restoration in HIV and SIV infection. *PLoS Pathog.* **12**, e1005349 (2016).

171. Sutherland, A. P. et al. IL-21 promotes CD8^+^ CTL activity via the transcription factor T-bet. *J. Immunol.* **190,** 3977-3984 (2013).

172. Stahl, M. et al. The forkhead transcription factor FoxO regulates transcription of p27Kip1 and Bim in response to IL-2. *J. Immunol.* **168,** 5024-5031 (2002).

173. Kratchmarov, R. et al. IRF4 couples anabolic metabolism to Th1 cell fate determination. *Immunohorizons* **1,** 156-161 (2017).

174. Zhang, Q. et al. LAG3 limits regulatory T cell proliferation and function in autoimmune diabetes. *Sci. Immunol.* **2,** eaah4569 (2017).

175. Whitmarsh, A. J. Regulation of gene transcription by mitogen-activated protein kinase signaling pathways. *Biochim. Biophys. Acta* **1773,** 1285-1298 (2007).

176. Waickman, A. T. & Powell, J. D. mTOR, metabolism, and the regulation of T-cell differentiation and function. *Immunol. Rev.* **249,** 43-58 (2012).

177. Man, K. & Kallies, A. Synchronizing transcriptional control of T cell metabolism and function. *Nat. Rev. Immunol.* **15,** 574-584 (2015).

178. Palazon, A. et al. An HIF-1α/VEGF-A axis in cytotoxic T cells regulates tumor progression. *Cancer Cell* **32,** 669-683.e5 (2017).

179. Yao, S. et al. Interferon regulatory factor 4 sustains CD8^+^ T cell expansion and effector differentiation. *Immunity* **39,** 833-845 (2013).

180. Wang, R. et al. The transcription factor Myc controls metabolic reprogramming upon T lymphocyte activation. *Immunity* **35,** 871-882 (2011).

181. Morrish, F. & Hockenbery, D. MYC and mitochondrial biogenesis. *Cold Spring Harb. Perspect. Med.* **4,** 014225(2014).

182. Ron-Harel, N. et al. Mitochondrial biogenesis and proteome remodeling promote one-carbon metabolism for T cell activation. *Cell Metab.* **24,** 104-117 (2016).

183. Wong, C., Chen, C., Wu, Q., Liu, Y. & Zheng, P. A critical role for the regulated wnt-myc pathway in naive T cell survival. *J. Immunol.* **194,** 158-167 (2015).

184. Brunner, T. et al. Expression of Fas ligand in activated T cells is regulated by c-Myc. *J. Biol. Chem.* **275,** 9767-9772 (2000).

185. Contini, P. et al. Apoptosis of antigen-specific T lymphocytes upon the engagement of CD8 by soluble HLA class I molecules is Fas ligand/Fas mediated: evidence for the involvement of p56lck, calcium calmodulin kinase II, and calcium-independent protein kinase C signaling pathways and for NF-kappaB and NF-AT nuclear translocation. *J. Immunol.* **175,** 7244-7254 (2005).

186. Yin, Y. et al. Impact of cytosine methylation on DNA binding specificities of human transcription factors. *Science* **356**, eaaj2239 (2017).

187. Oestreich, K. J., Yoon, H., Ahmed, R. & Boss, J. M. NFATc1 regulates PD-1 expression upon T cell activation. *J. Immunol.* **181,** 4832-4839 (2008).

188. Chuvpilo, S. et al. Autoregulation of NFATc1/A expression facilitates effector T cells to escape from rapid apoptosis. *Immunity* **16,** 881-895 (2002).

189. Mognol, G. P., Carneiro, F. R., Robbs, B. K., Faget, D. V. & Viola, J. P. Cell cycle and apoptosis regulation by NFAT transcription factors: new roles for an old player. *Cell Death Dis.* **7,** e2199 (2016).

190. Gibson, H. M. et al. Induction of the CTLA-4 gene in human lymphocytes is dependent on NFAT binding the proximal promoter. *J. Immunol.* **179,** 3831-3840 (2007).

191. Vaeth, M. & Feske, S. NFAT control of immune function: new frontiers for an abiding trooper. Version 1. *F1000Res.* **7,** 260 (2018).

192. van Rietschoten, J. G. et al. Silencer activity of NFATc2 in the interleukin-12 receptor beta 2 proximal promoter in human T helper cells. *J. Biol. Chem.* **276,** 34509-34516 (2001).

193. Stauss, D. et al. The transcriptional coactivator Bob1 promotes the development of follicular T helper cells via Bcl6. *EMBO J.* **35,** 881-898 (2016).

194. Hui, E. et al. T cell costimulatory receptor CD28 is a primary target for PD-1-mediated inhibition. *Science* **355,** 1428-1433 (2017).

195. LeBleu, V. S. et al. PGC-1α mediates mitochondrial biogenesis and oxidative phosphorylation in cancer cells to promote metastasis. *Nat. Cell Biol.* **16,** 992-1003 (2014).

196. Scarpulla, R. C. Metabolic control of mitochondrial biogenesis through the PGC-1 family regulatory network. *Biochim. Biophys. Acta* **1813,** 1269-1278 (2011).

197. Hand, T. W. et al. Differential effects of STAT5 and PI3K/AKT signaling on effector and memory CD8 T-cell survival. *Proc. Natl. Acad. Sci. U S A* **107,** 16601-16606 (2010).

198. D'Souza, W. N., Chang, C. F., Fischer, A. M., Li, M. & Hedrick, S. M. The Erk2 MAPK regulates CD8 T cell proliferation and survival. *J. Immunol.* **181,** 7617-7629 (2008).

199. Chen, Y. & Yu, D. TCF-1 at the Tfh and Th1 Divergence. *Trends Immunol.* **36,** 758-760 (2015).

200. Wu, T. et al. The TCF1-Bcl6 axis counteracts type I interferon to repress exhaustion and maintain T cell stemness. *Sci. Immunol.* **1,** eaai8593 (2016).

201. Chauvin, J. M. et al. TIGIT and PD-1 impair tumor antigen-specific CD8^+^ T cells in melanoma patients. *J. Clin. Invest.* **125,** 2046-2058 (2015).

202. Kurtulus, S. et al. TIGIT predominantly regulates the immune response via regulatory T cells. *J. Clin. Invest.* **125,** 4053-4062 (2015).

203. Sabins, N. C. et al. TIM-3 engagement promotes effector memory T cell differentiation of human antigen-specific CD8 T cells by activating mTORC1. *J. Immunol.* **199,** 4091-4102 (2017).

204. Dawicki, W. & Watts, T. H. Expression and function of 4-1BB during CD4 versus CD8 T cell responses in vivo. *Eur. J. Immunol.* **34,** 743-751 (2004).

205. Cannons, J. L. et al. 4-1BB ligand induces cell division, sustains survival, and enhances effector function of CD4 and CD8 T cells with similar efficacy. *J. Immunol.* **167,** 1313-1324 (2001).

206. Ban, Y. H. et al. miR-150-mediated Foxo1 regulation programs CD8^+^ T cell differentiation. *Cell Rep.* **20,** 2598-2611 (2017).

207. Kakaradov, B. et al. Early transcriptional and epigenetic regulation of CD8^+^ T cell differentiation revealed by single-cell RNA sequencing. *Nat. Immunol.* **18,** 422-432 (2017).

208. Ghoneim, H. E. et al. De novo epigenetic programs inhibit PD-1 blockade-mediated T cell rejuvenation. *Cell* **170,** 142-157.e19 (2017).

**Supplementary** **Fig. S1.** Diverse mechanisms can lead to a failure of CD8^+^ T cells to respond to tumors. In the scenarios to the left of the figure, CD8^+^ T cells are either not activated (e.g. due to a dearth of antigens or spatial exclusion of T cells), or they may be unable to respond due to extrinsic suppression. In senescence, anergy and exhaustion on the other hand, CD8^+^ T cells are activated but hypofunctional.

CD8^+^ T Cell failure to respond

Spatial exclusion

- Neutrophils
- Matrix architecture
- Chemokines, …

Cellular suppression

- Tregs
- MDSCs
- Pericyte dysfunction
- …

Hypofunctionality

Senescence

Anergy

**Exhaustion**

Metabolic/

nutrient/O_2_

deprivation

No antigen

(or MHC 1 loss)

Iatrogenic immune suppression


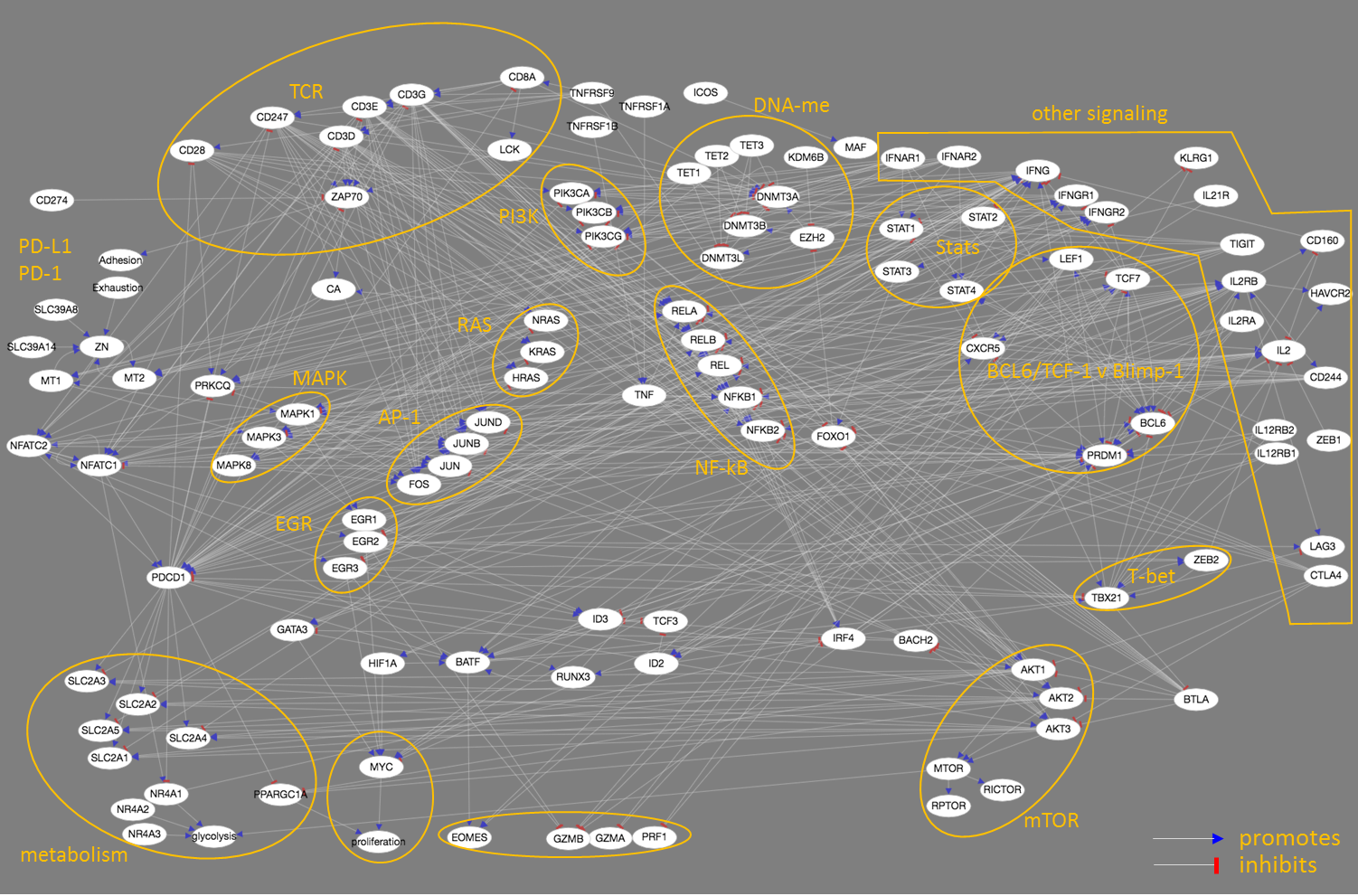
**Supplementary** **Fig. S2.** Literature-based network of molecular interactions underlying CD8^+^ T cell exhaustion (TCE), comprising 505 interactions and 113 genes (119 nodes). For simplicity, each node in the network represents the overall activity of a gene *and* its product(s). Edges (connecting lines) represent reported interactions. Edges ending in a blue arrowhead indicate that the source node ‘promotes’ the target node. Edges ending in red bars denote repression. Orange labels and bounding boxes mark groups of related genes/gene products.

**Supplementary** **Fig. S3.** Superposition of expression data onto the TCE network highlights time/condition dependence of interactions. Node colors indicate mRNA expression relative to naïve CD8^+^ cells (see color bar at top left). Red edges are inhibitory. Blue edges are promoting. Edge thickness indicates the fraction of replicates in which the source and target gene expression are concordant with the sense of edge (see key at bottom right). Day 5, acute infection data from GSE89307, Schietinger lab, 2017.


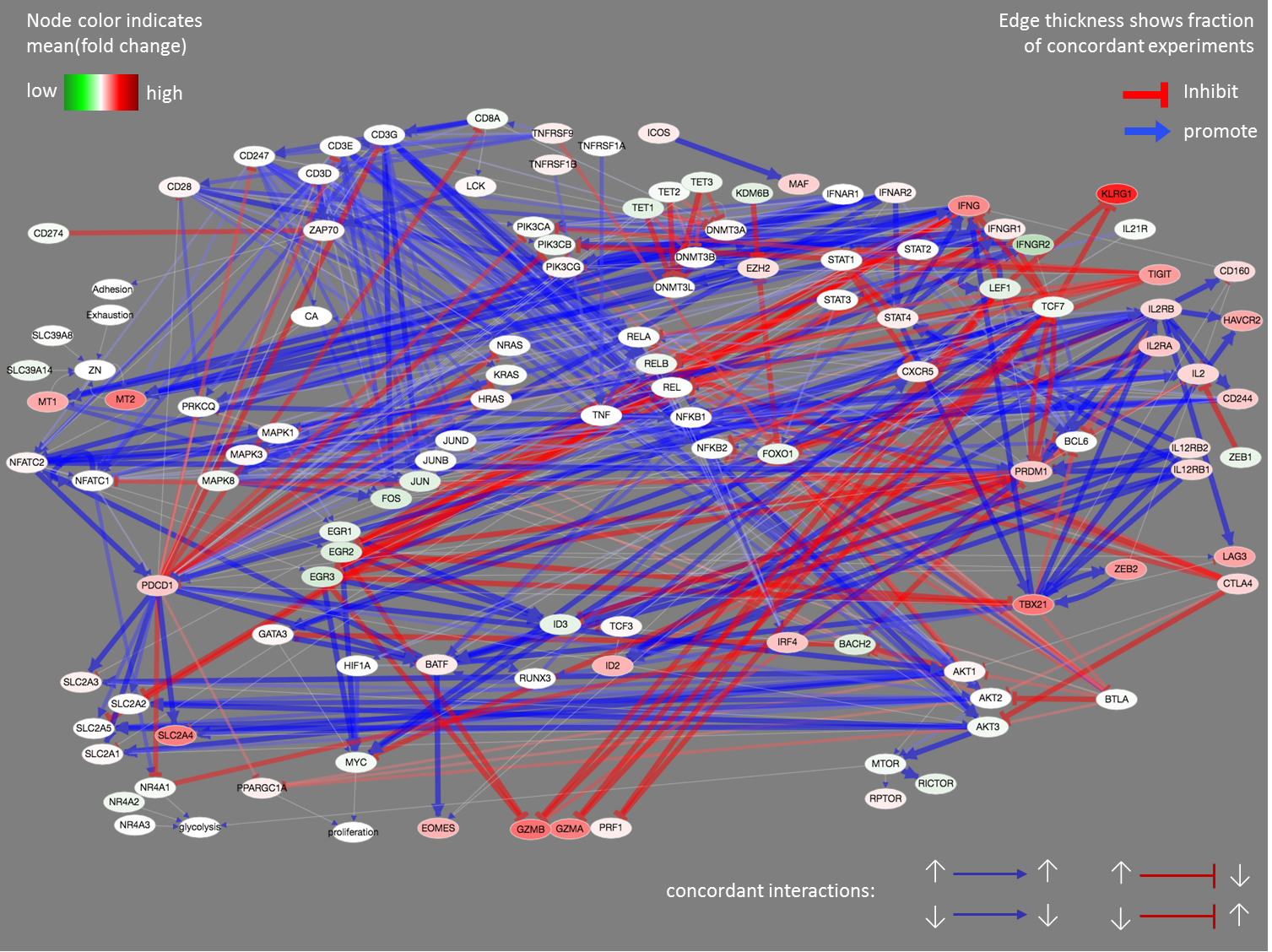


Concordant interactions:

Low

High

Promote

**Supplementary** **Fig. S4**. Superposition of expression data onto the TCE network highlights time/condition dependence of interactions. Node colors indicate mRNA expression relative to naïve CD8^+^ cells (see color bar at top left). Red edges are inhibitory. Blue edges are promoting. Edge thickness indicates the fraction of replicates in which the source and target gene expression are concordant with the sense of edge (see key at bottom right). Day 7, acute infection data from GSE89307, Schietinger lab, 2017.


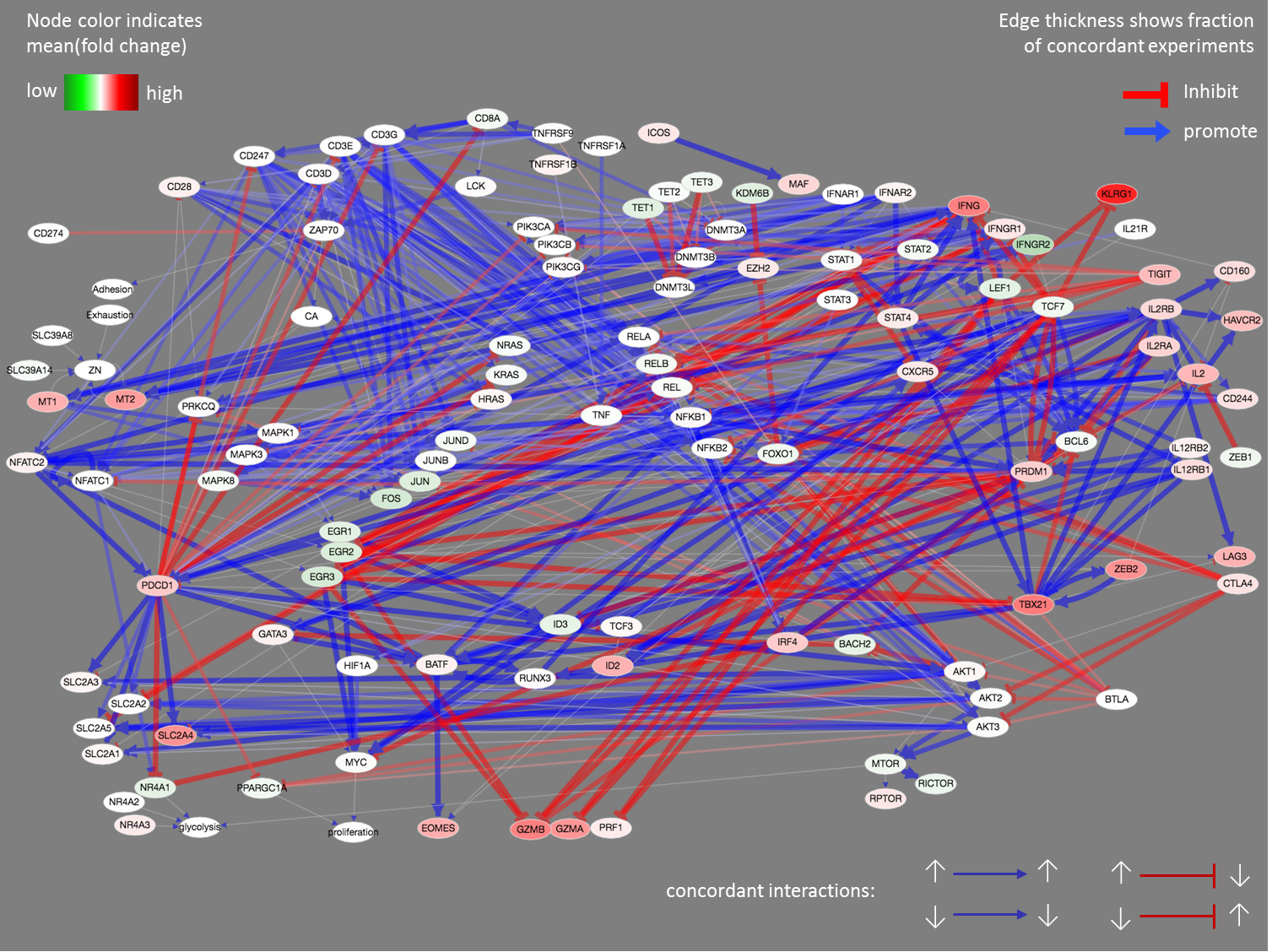


High

Low

Promote

Concordant interactions:

**Supplementary** **Fig. S5**. Superposition of expression data onto the TCE network highlights time/condition dependence of interactions. Node colors indicate mRNA expression relative to naïve CD8^+^ cells (see color bar at top left). Red edges are inhibitory. Blue edges are promoting. Edge thickness indicates the fraction of replicates in which the source and target gene expression are concordant with the sense of edge (see key at bottom right). Day 5, tumor data from GSE89307, Schietinger lab, 2017.

**
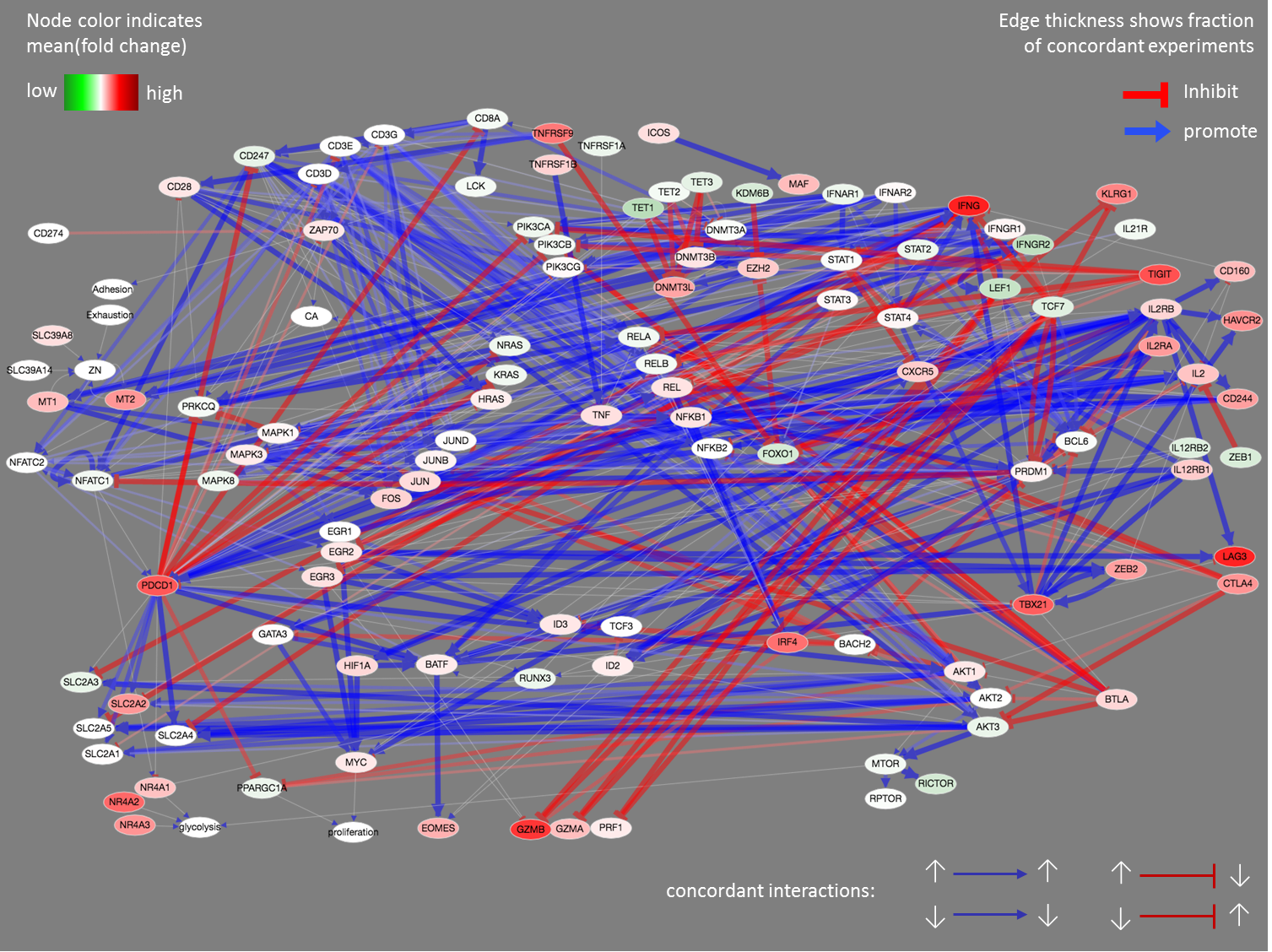
**

High

Low

Concordant interactions:

Promote

**Supplementary** **Fig. S6**. Superposition of expression data onto the TCE network highlights time/condition dependence of interactions. Node colors indicate mRNA expression relative to naïve CD8^+^ cells (see color bar at top left). Red edges are inhibitory. Blue edges are promoting. Edge thickness indicates the fraction of replicates in which the source and target gene expression are concordant with the sense of edge (see key at bottom right). Day 7, tumor data from GSE89307, Schietinger lab, 2017.


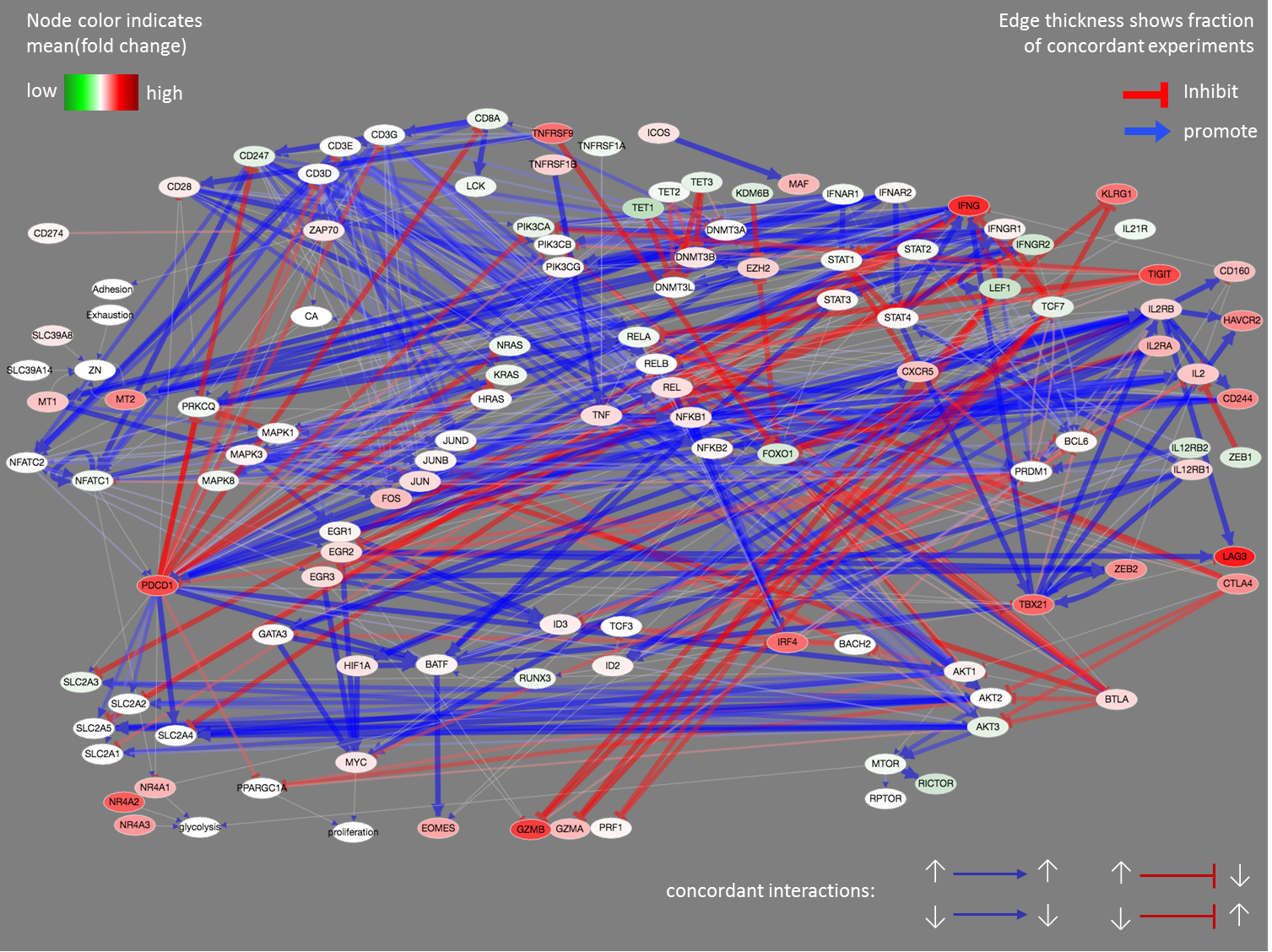


High

Low

Concordant interactions:

Promote

**Supplementary** **Fig. S7**. Superposition of expression data onto the TCE network highlights time/condition dependence of interactions. Node colors indicate mRNA expression relative to naïve CD8^+^ cells (see color bar at top left). Red edges are inhibitory. Blue edges are promoting. Edge thickness indicates the fraction of replicates in which the source and target gene expression are concordant with the sense of edge (see key at bottom right). Day 14, tumor data from GSE89307, Schietinger lab, 2017.


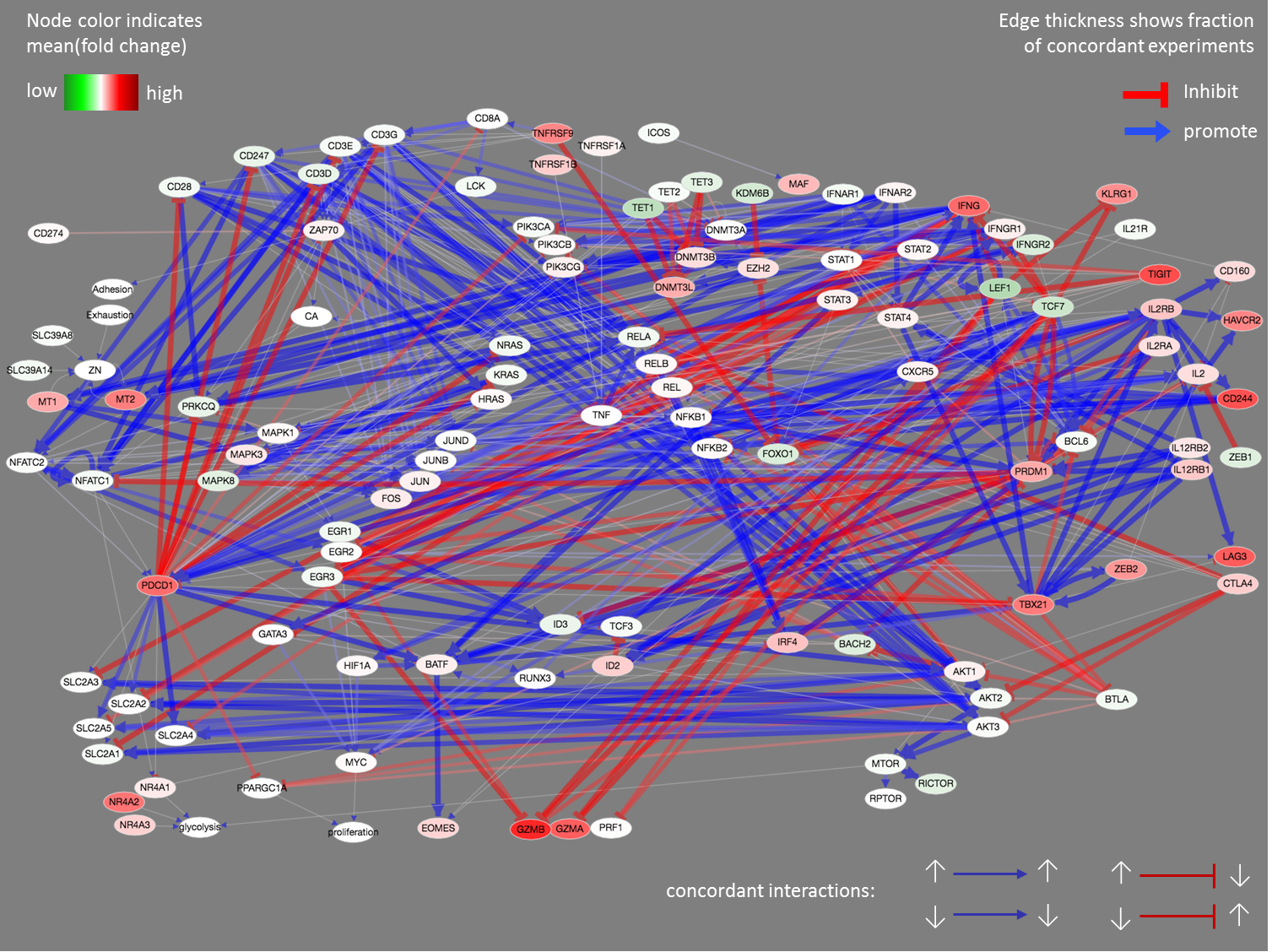


Concordant interactions:

Promote

Low

High

**Supplementary** **Fig. S8**. Superposition of expression data onto the TCE network highlights time/condition dependence of interactions. Node colors indicate mRNA expression relative to naïve CD8^+^ cells (see color bar at top left). Red edges are inhibitory. Blue edges are promoting. Edge thickness indicates the fraction of replicates in which the source and target gene expression are concordant with the sense of edge (see key at bottom right). Day 21, tumor data from GSE89307, Schietinger lab, 2017.


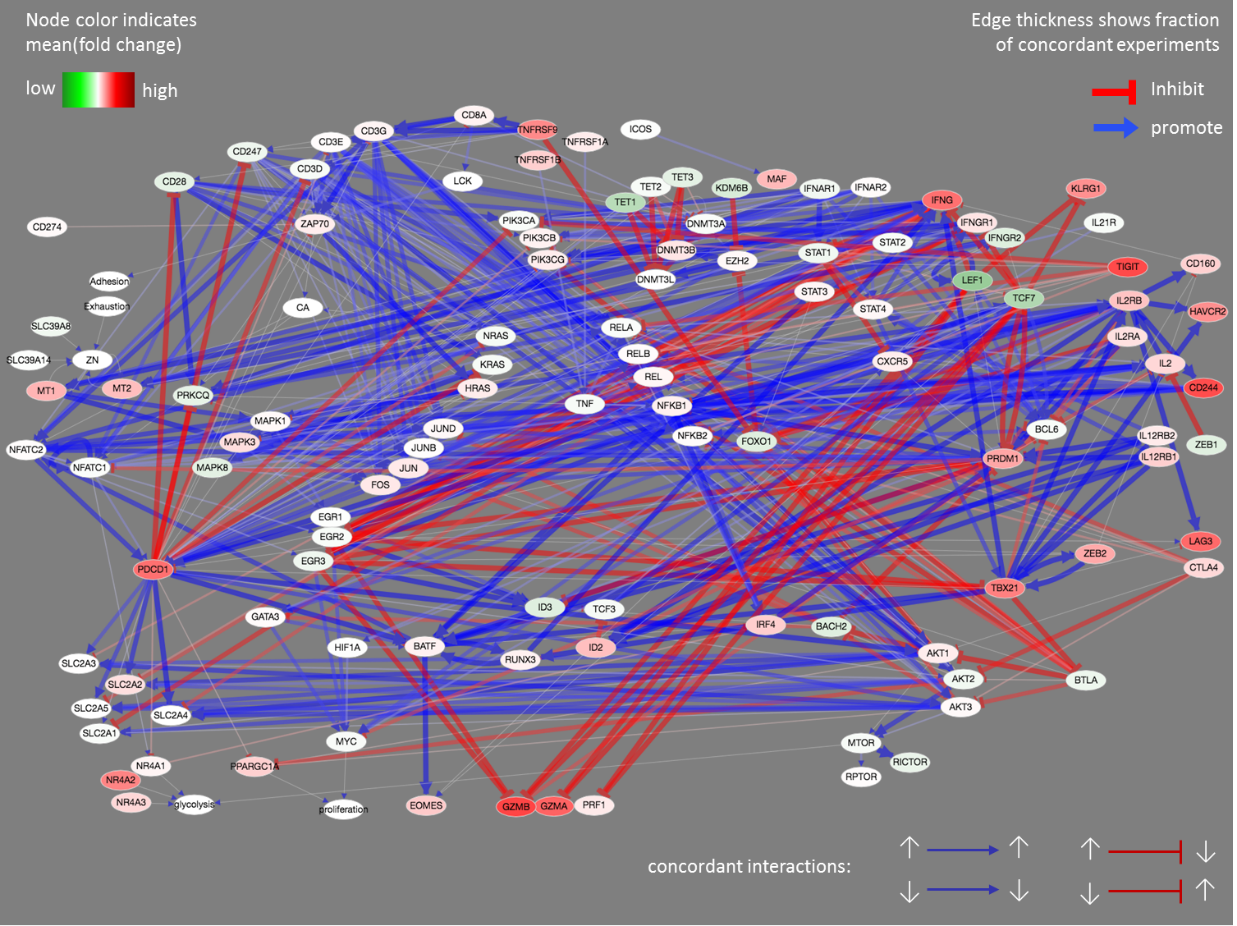


High

Low

Promote

Concordant interactions:

**Supplementary** **Fig. S9**. Genes and interactions in our TCE network are consistent with experimental data. The heatmap at left shows edges (rows) in which expression fold change with respect to naïve cells in GSE89307 is concordant (red) or not (blue) with the sense of the edge. 17 TCE-network edges (3.6% of 478 gene-gene edges) were not concordant in any condition, listed below in the format ‘source:target:edgeSense’:

[1] AKT1:MTOR:1 BATF:IRF4:-1 CD160:IFNG:-1 CD160:IL2:-1

[5] CTLA4:AKT1:-1 DNMT3A:DNMT3A:-1 FOXO1:PDCD1:1 IFNG:SLC2A4:-1

[9] LEF1:EOMES:1 TBX21:CD160:-1 TBX21:LAG3:-1 TBX21:PDCD1:-1

[13] TCF7:EOMES:1 TCF7:IFNGR2:-1 TET1:PDCD1:1 TET3:PDCD1:1

[17] ZEB2:IL2:-1

Right panel: using the same data and 500,000 randomly assigned edges, concordance scores of 1 occur at a false discovery rate of ~15%.

0

Frequency

Edge concordance score

False discovery rate = 14.8% in 500,000 randomizations


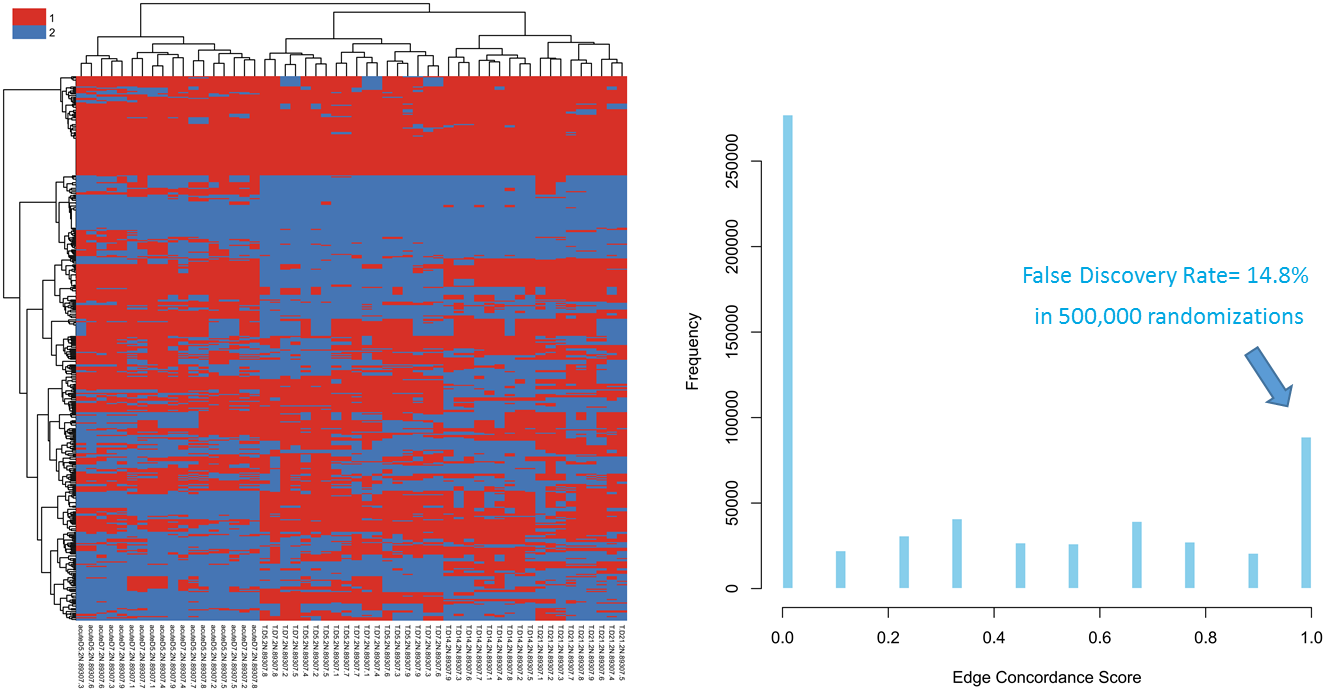


**Supplementary**
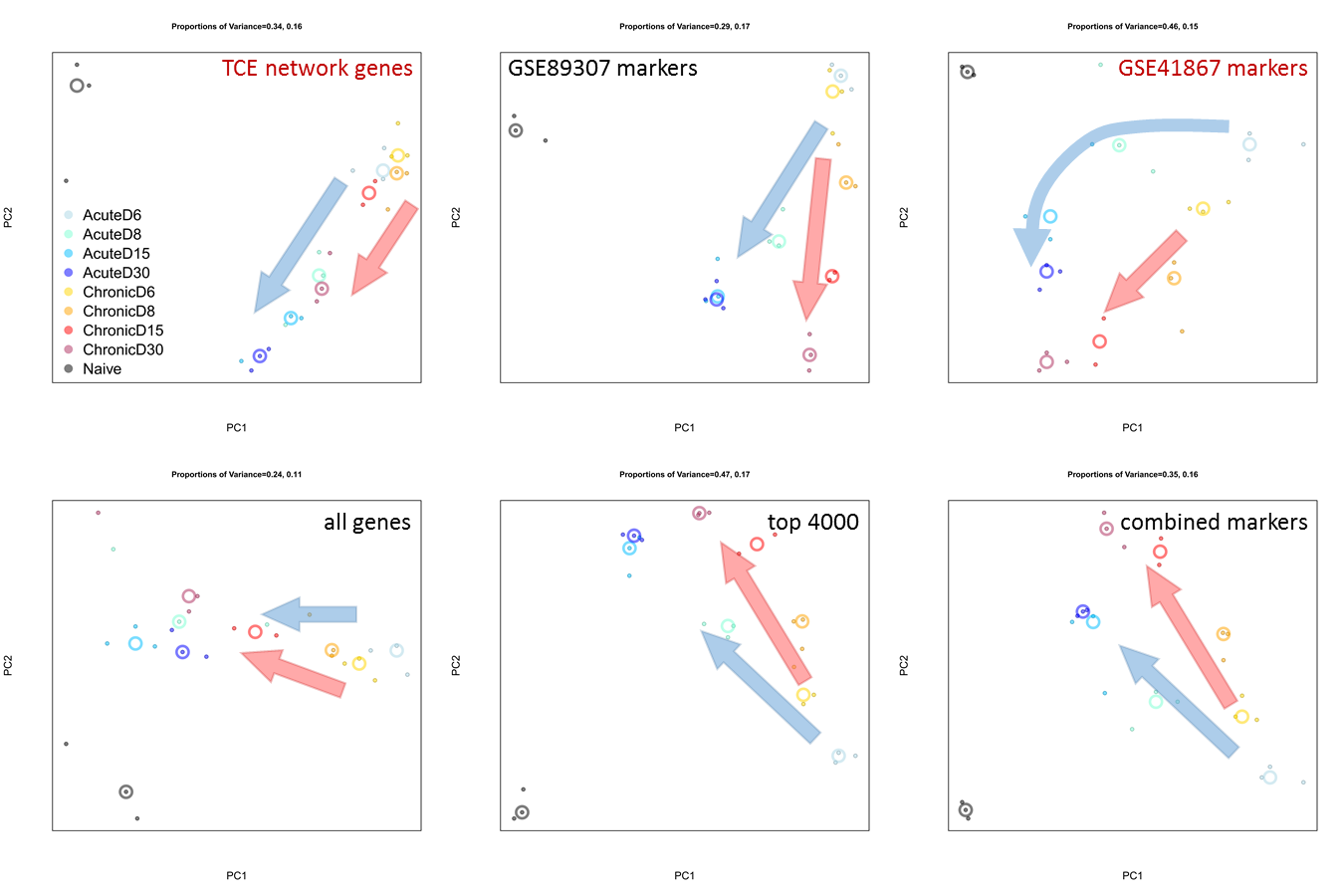
**Fig. S10**. The TCE network genes track CD8^+^ evolution during both acute and chronic stimulation. Example PCA plots showing data from GSE89307 (mouse liver tumor CD8^+^ tumor-infiltrating lymphocytes). The top row shows principal component analysis (PCA) plots using the TCE network genes (left) and 2 published gene sets, as indicated. In all 3 plots, acute (shades of blue) and chronic (yellow to red) state transitions have similar trajectories and are monotonic with respect to time. The lower 3 plots confirm these observations using 3 alternate gene sets, as indicated.

Top 4000

All genes

Combined markers

GSE41867 markers

GSE89307 markers

TCE network genes

**Supplementary** **Fig. S11**. Acute and chronic stimulation of CD8^+^ cells show similar metabolic profiles. Metabolic pathway activity scores were generated as described in Methods. The heatmap on the left is for GSE89307 (mouse liver tumor CD8^+^ tumor-infiltrating lymphocytes). Columns are CD8^+^ cells in various states: N = naïve. E5 and E7 = acute infection at days 5 and 7. TDx = tumor CD8^+^ cells at day ‘x’, as indicated. Areas overlaid in gray show memory cell states. The heat map on the right shows metabolic pathway activities of acutely stimulated CD8^+^ cells for GSE15907 (the Immunological Genome Project). ‘Nve’ marks naïve cells. ‘VSV’ = vesicular stomatitis virus. ‘Lis’ = *Listeria monocytogenes*. Days and hours post-infection are indicated by ‘d’ and ‘hr’.


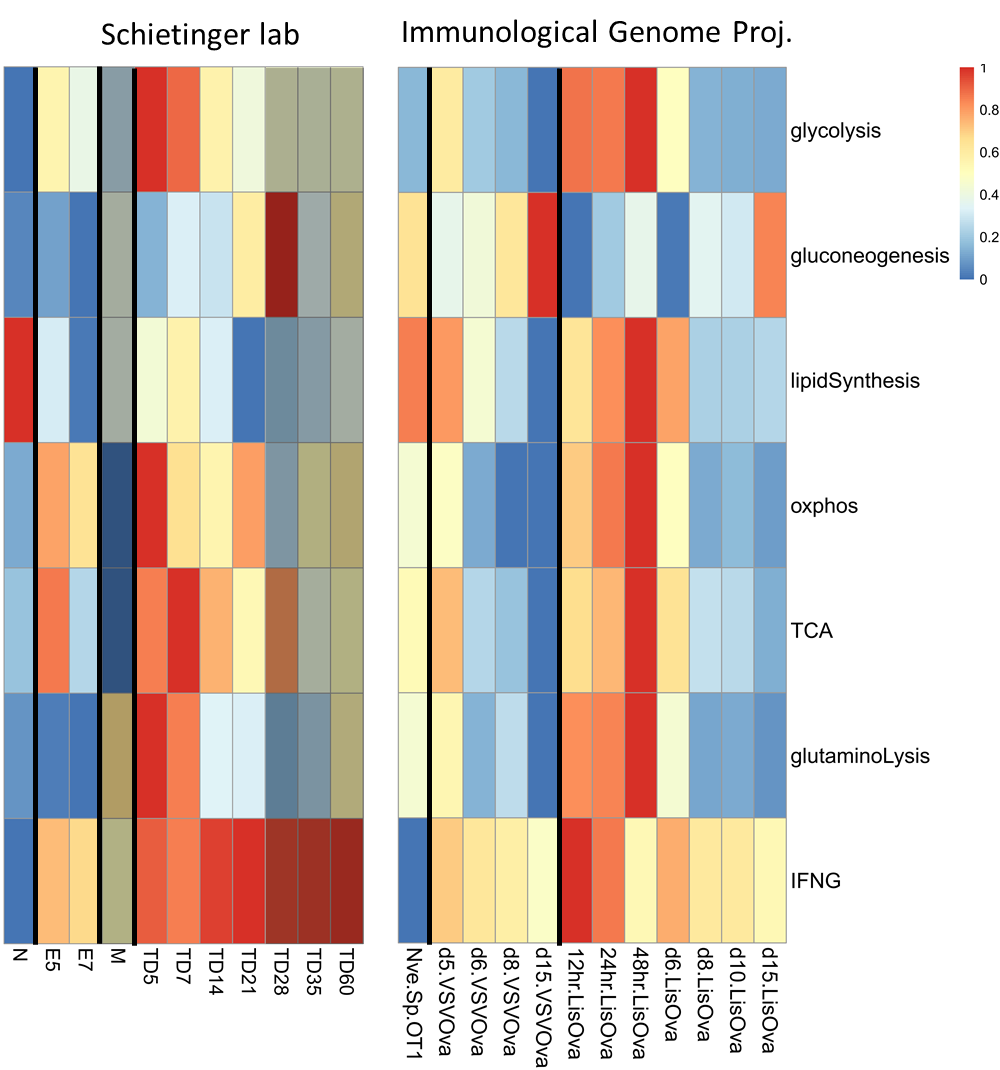


Lipid Synthesis

IFN-γ

TCA

GlutaminoLysis

Oxphos

Gluconeogenesis

Glycolysis

Immunological Genome Project

Schietinger lab

**Supplementary** **Fig. S12**. Changes in metabolic gene activity track CD8^+^ TCE network state changes during both acute and chronic CD8^+^ T cell stimulation. Principal component analysis plots for 2 data sets are shown as examples. Arrows indicate the direction of change over time. See Methods for a list of the metabolic marker genes used.


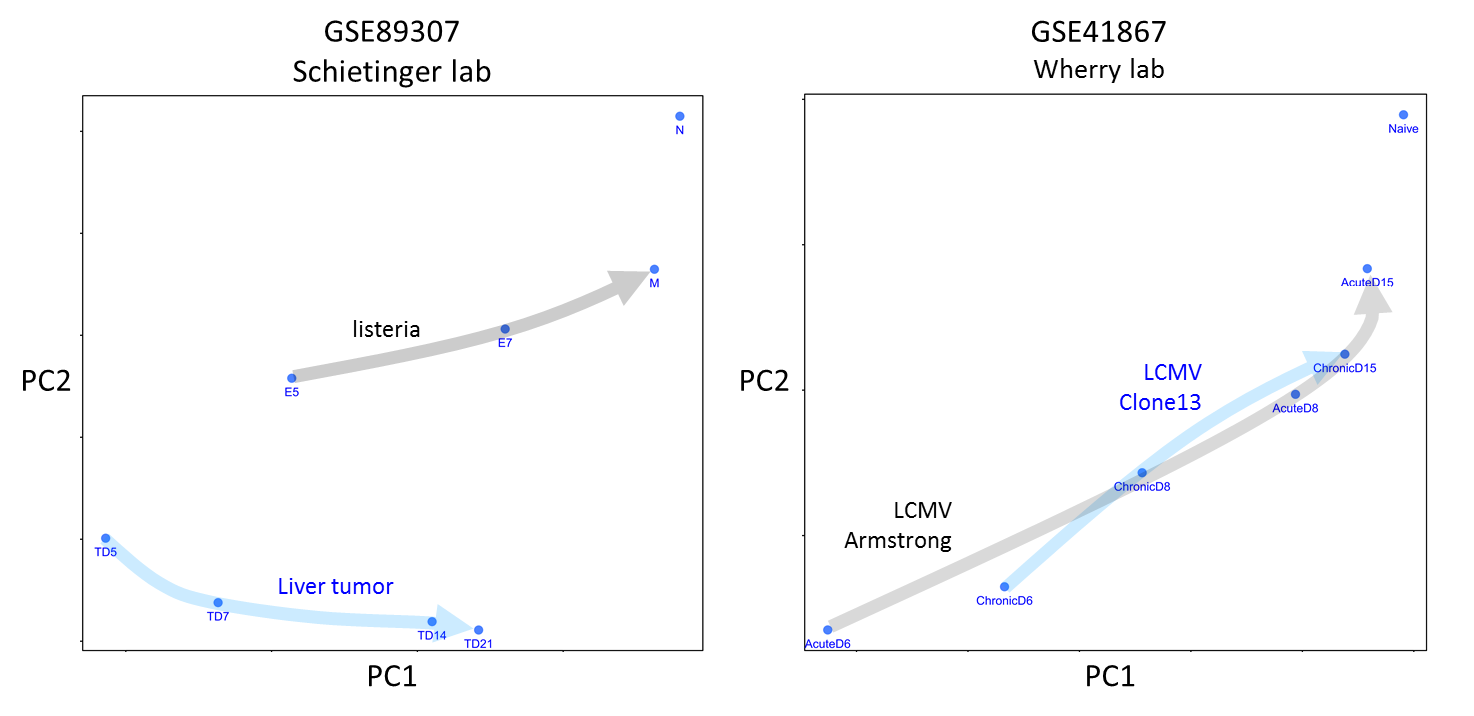


LCMV
Armstrong

LCMV
Clone13

Liver tumor

Listeria

PC2

PC1

PC1

PC2

GSE89307
Schietinger lab

GSE41867
Wherry lab

**Supplementary** **Fig. S13**. Co-expression gene clusters for GSE89307 (Schietinger lab mouse liver tumor CD8^+^ T cells). Example clusters showing up(down)-regulation between days 7 and 14 following tumor initiation (‘early’ and ‘late’ activity phases) are highlighted in blue (yellow) background.


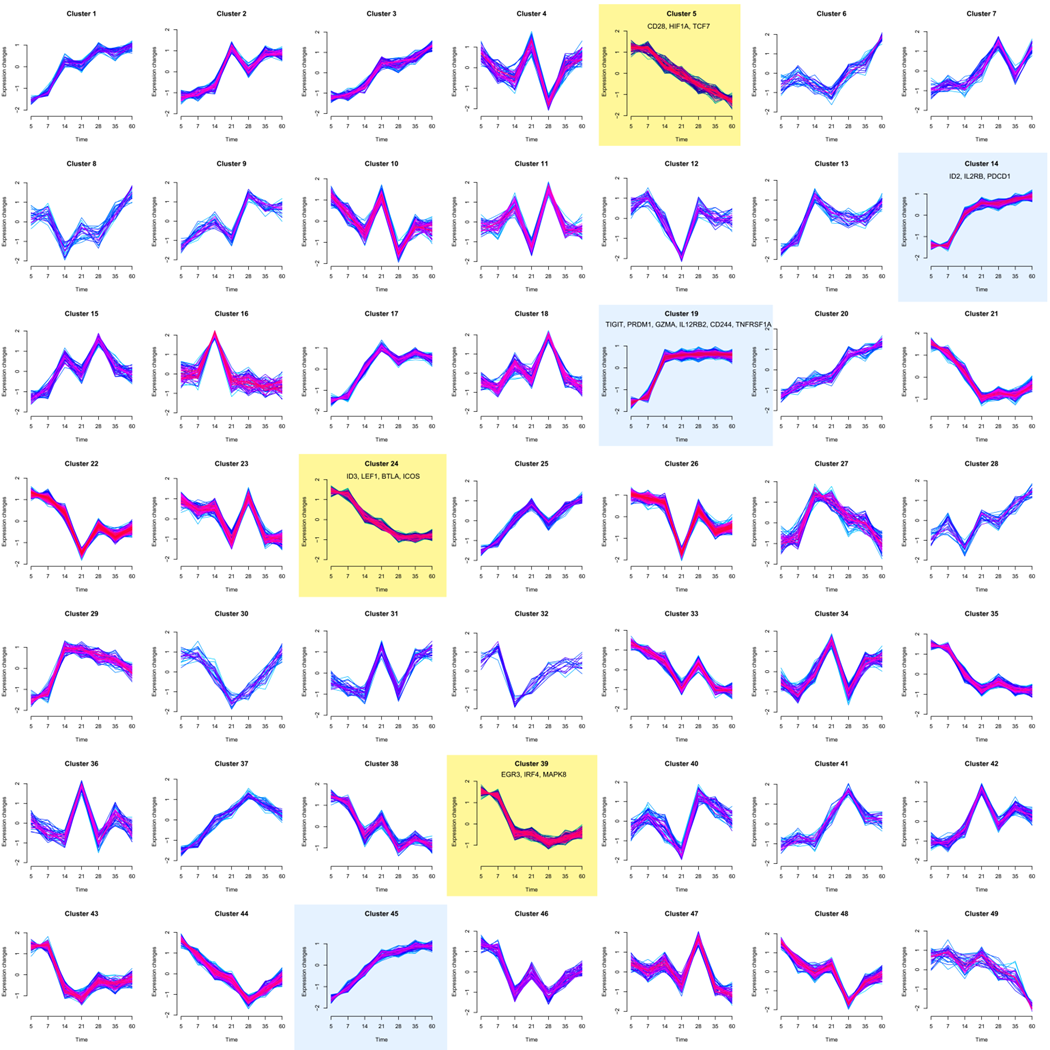


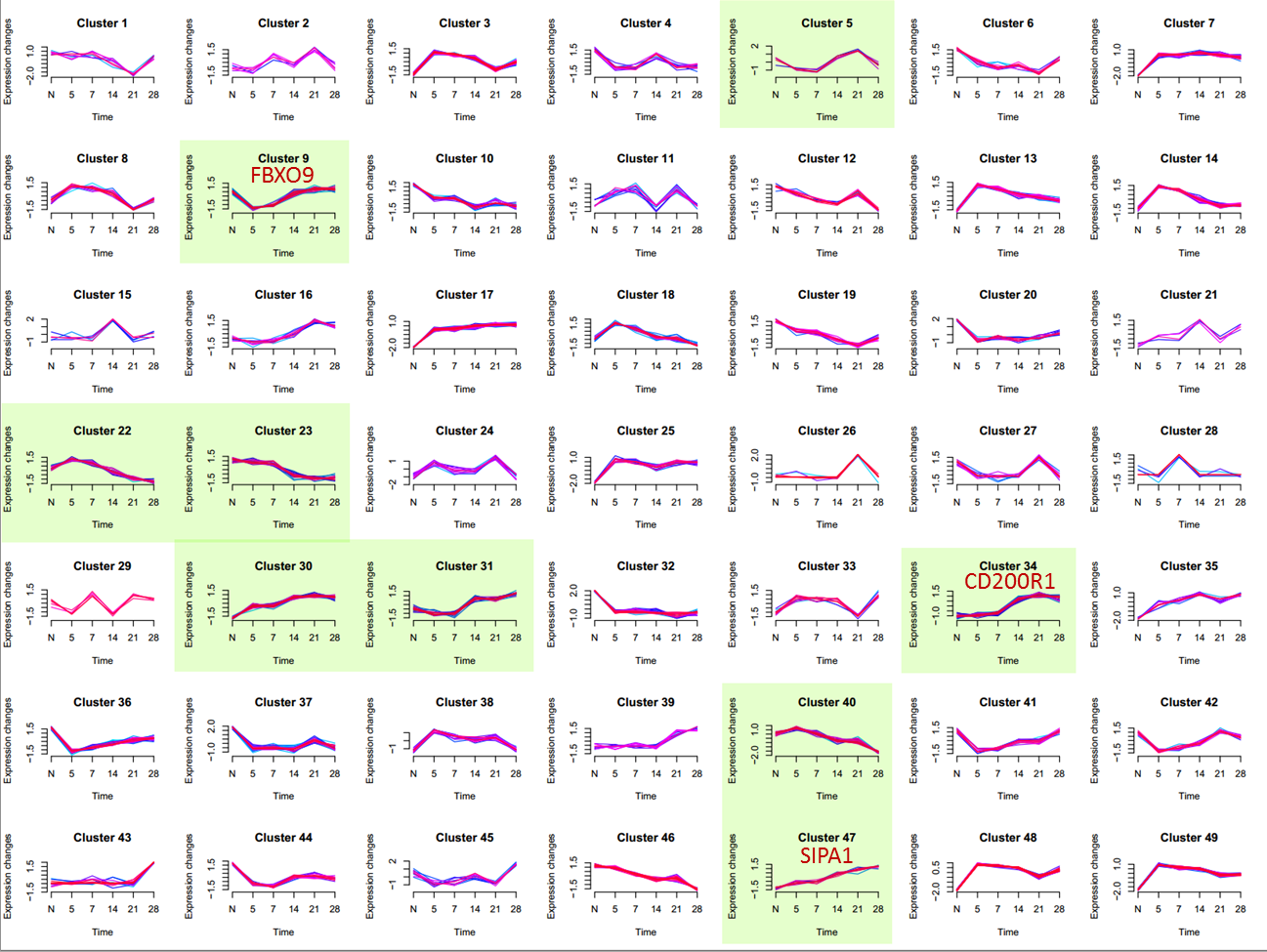
**Supplementary** **Fig. S14**. Expression clusters of *metabolic* genes in tumor-infiltrating lymphocytes (GSE89307, Schietinger lab, 2017). Green background marks genes changing between days 7 and 14 (‘early’ and ‘late’ phases). Clusters containing the 3 genes discussed in Supplementary Fig. 16 are marked in red.

**Supplementary** **Fig. S15.** Expression clusters of *metabolic* genes in chronic LCMV (GSE41867, Wherry lab, 2012). Green background marks genes changing between days 6 and 15 (‘early’ and ‘late’ phases). Clusters containing the 3 genes discussed in Supplementary Fig. 16 are marked in red.


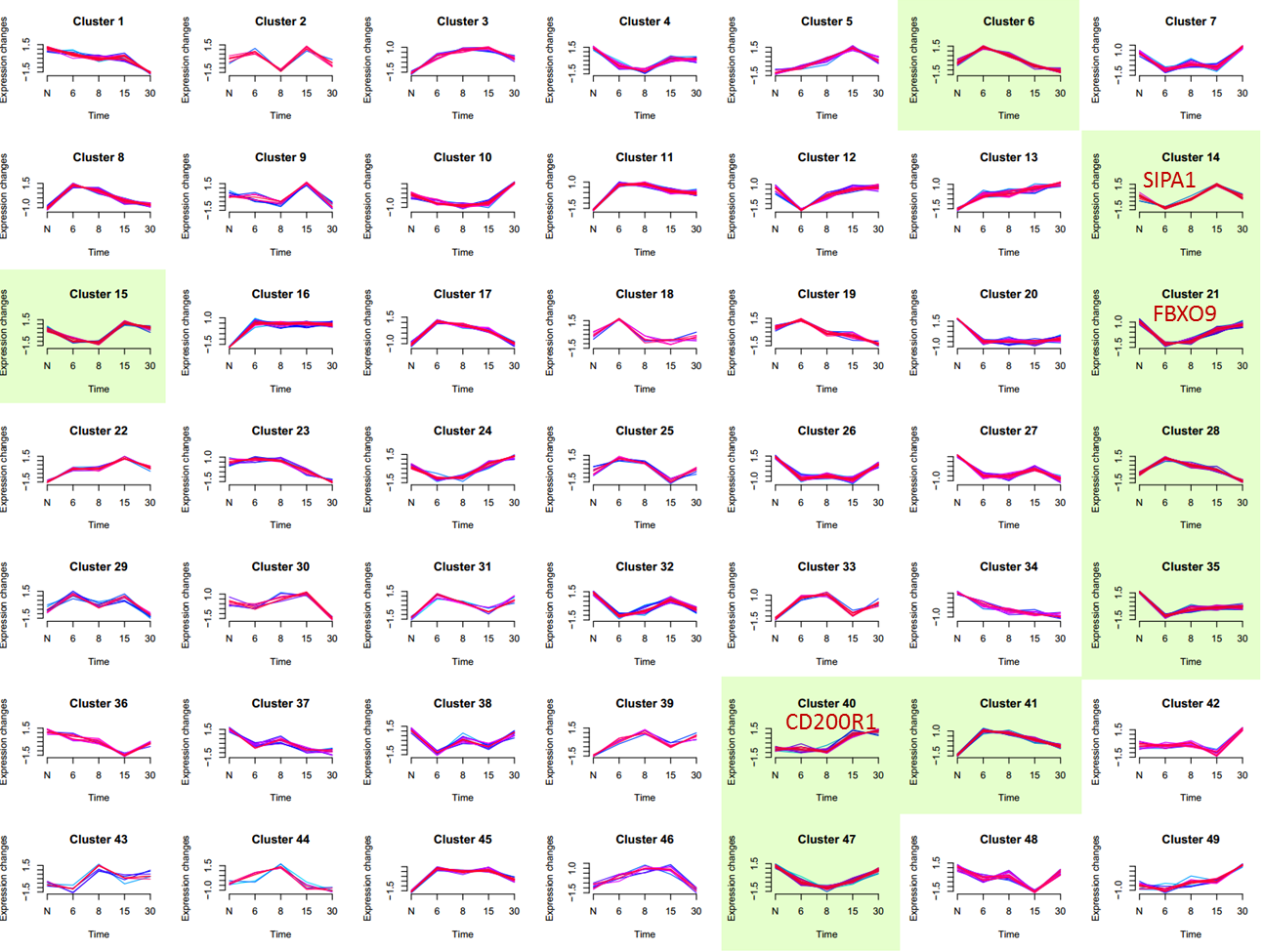


**Supplementary** **Fig. S16**. Active-late, chronic-only, metabolic-cluster genes that are differently clustered in acute response (all 3 genes are TP53 regulated). CD200R1 is known to repress ERK1/2 and IFNγ^1^, and confers tolerance^2^. FBXO9 suppresses mTORC1 and cell proliferation^3^. SIPA1 suppresses RAS signaling and proliferation^4^. Of the 3 genes, only CD200R1 shows high absolute fold changes in both chronic infection and tumor settings, but not in acute infection (right hand panels).

**
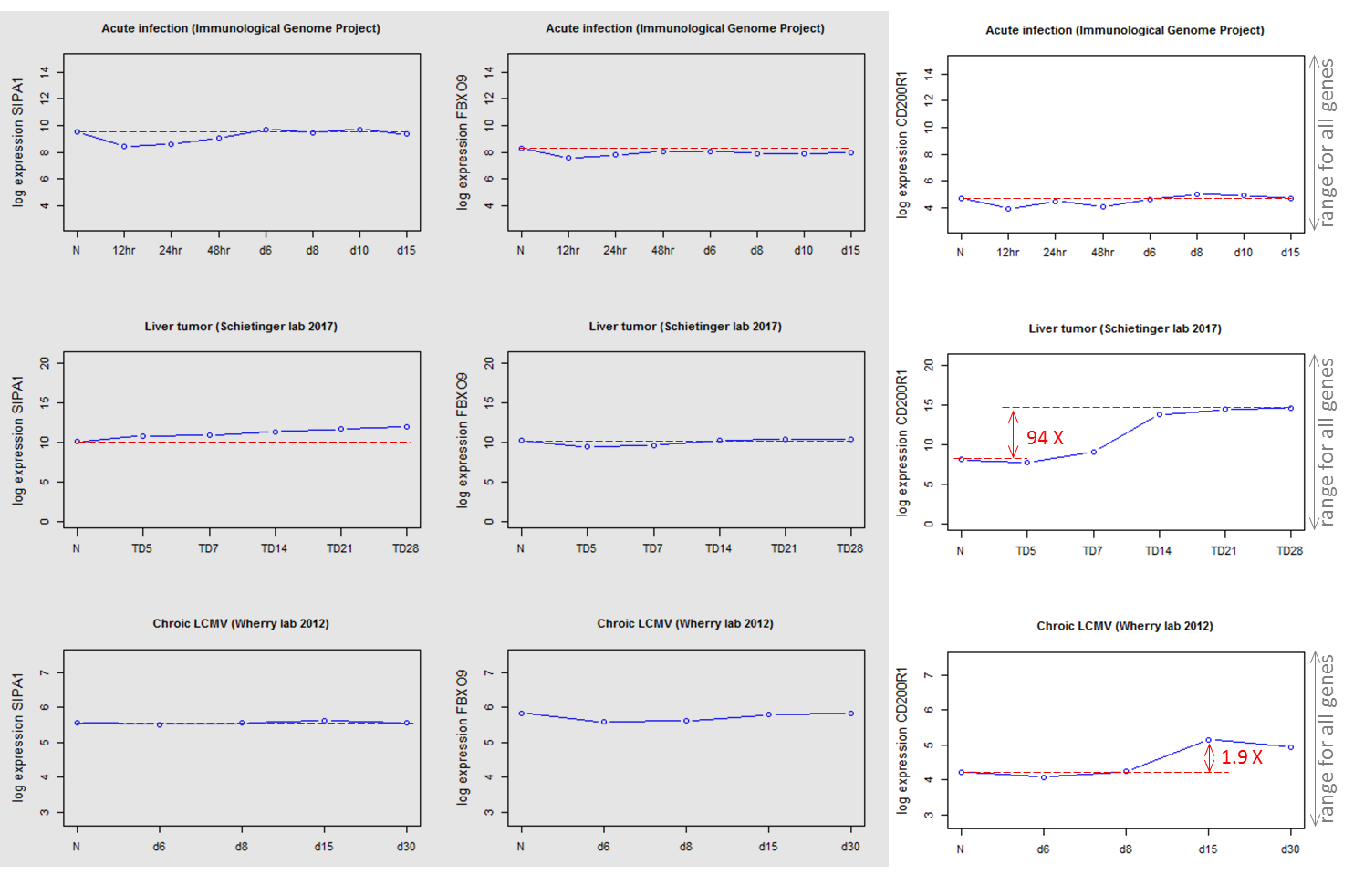
**

Range for all genes

Range for all genes

Range for all genes

**Chronic LCMV (Wherry lab 2012)**

**Chronic LCMV (Wherry lab 2012)**

**Chronic LCMV (Wherry lab 2012)**

**Supplementary Fig. S17**. Initial (starting) state of an example Boolean logic model booleannet (https://github.com/ialbert/booleannet)^5^ (see Methods for model equations). Red nodes are on. The remaining nodes are off. PathwayMapper (http://pathwaymapper.org/)^6^.


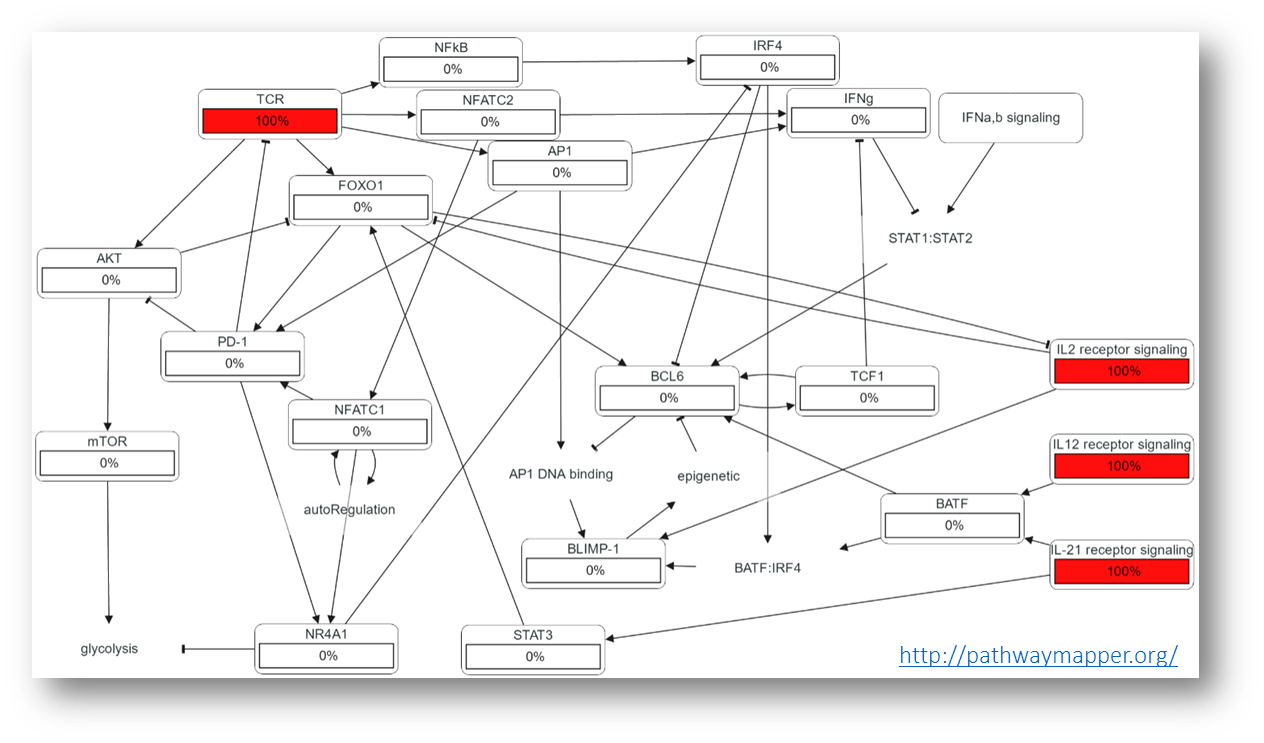


**Supplementary Fig. S18**. Early acute response state of the example Boolean logic model booleannet (https://github.com/ialbert/booleannet)^5^. Red nodes are on. The remaining nodes are off. PathwayMapper (http://pathwaymapper.org/)^6^.


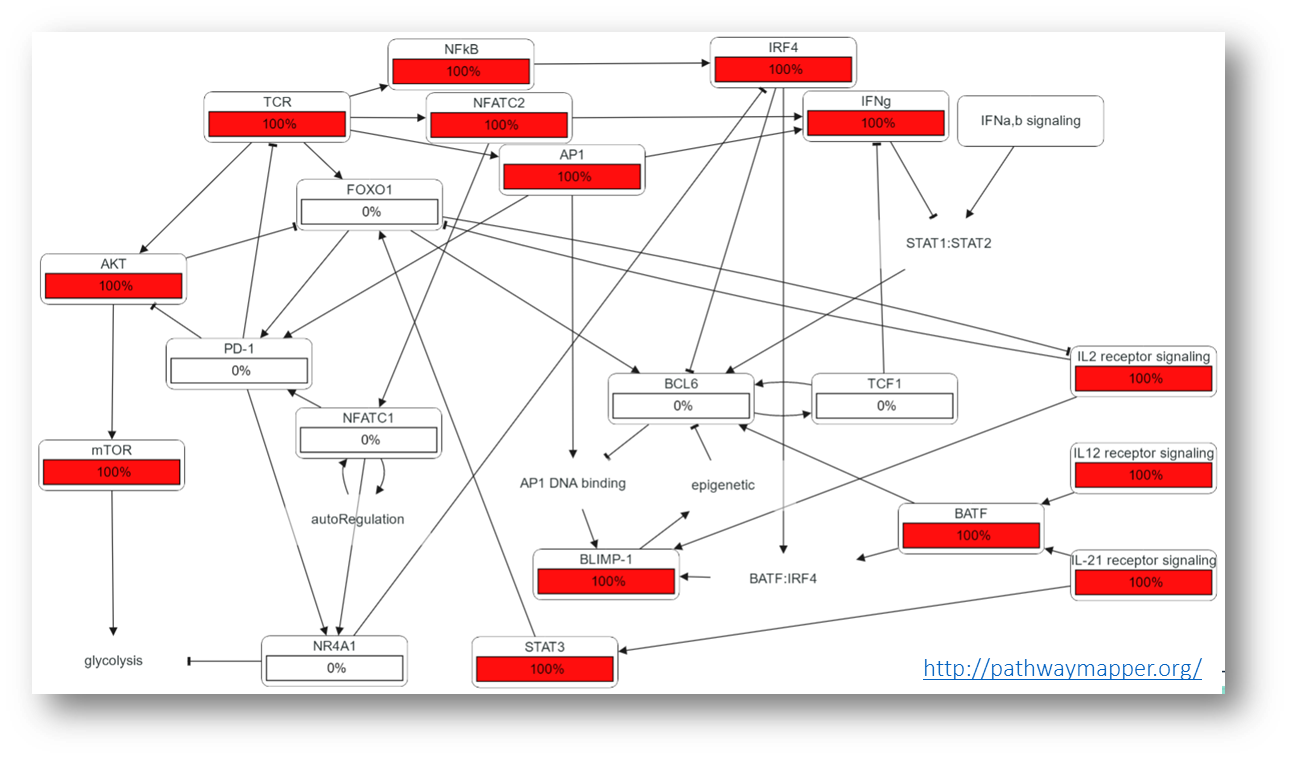


**Supplementary Fig. S19**. Late acute response state of an example Boolean logic model booleannet (https://github.com/ialbert/booleannet)^5^. Red nodes are on. The remaining nodes are off. PathwayMapper (http://pathwaymapper.org/)^6^.


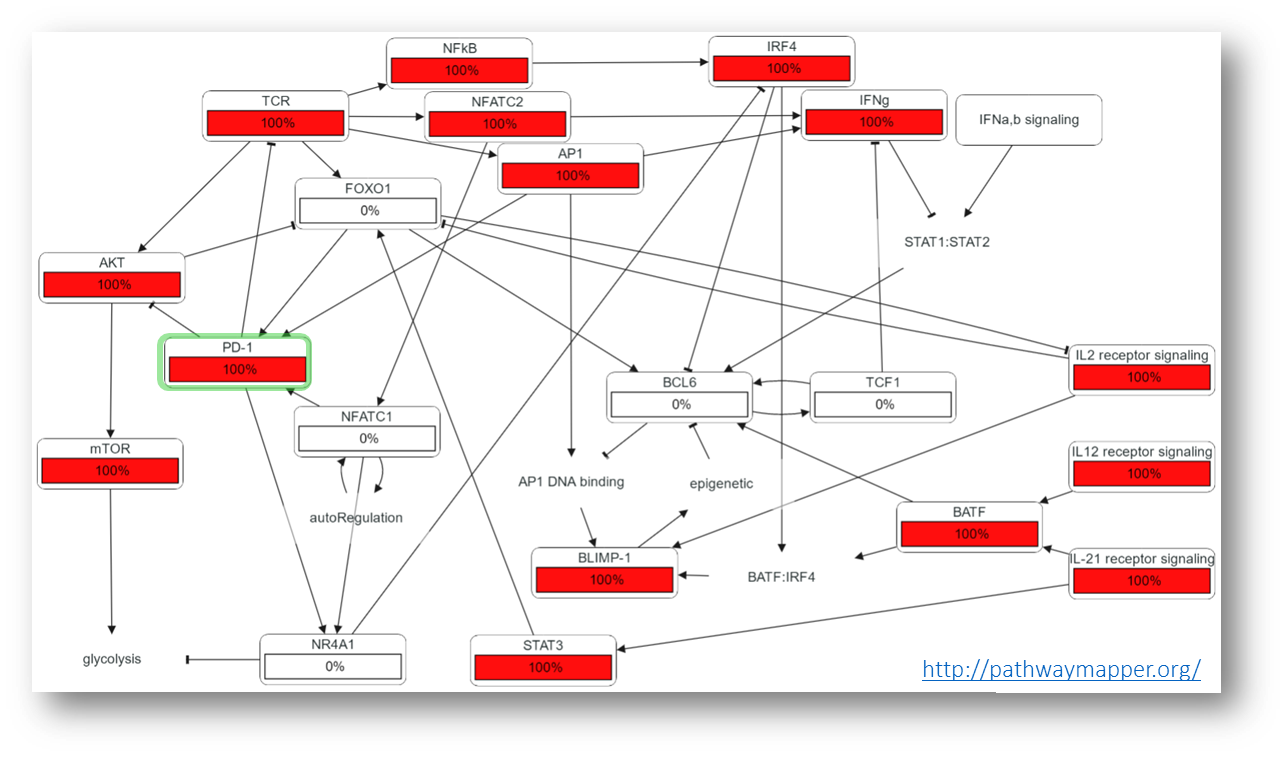


**Supplementary Fig. S20**. Terminal exhaustion state of an example Boolean logic model booleannet (https://github.com/ialbert/booleannet)^5^. Red nodes are on. The remaining nodes are off. PathwayMapper (http://pathwaymapper.org/)^6^.


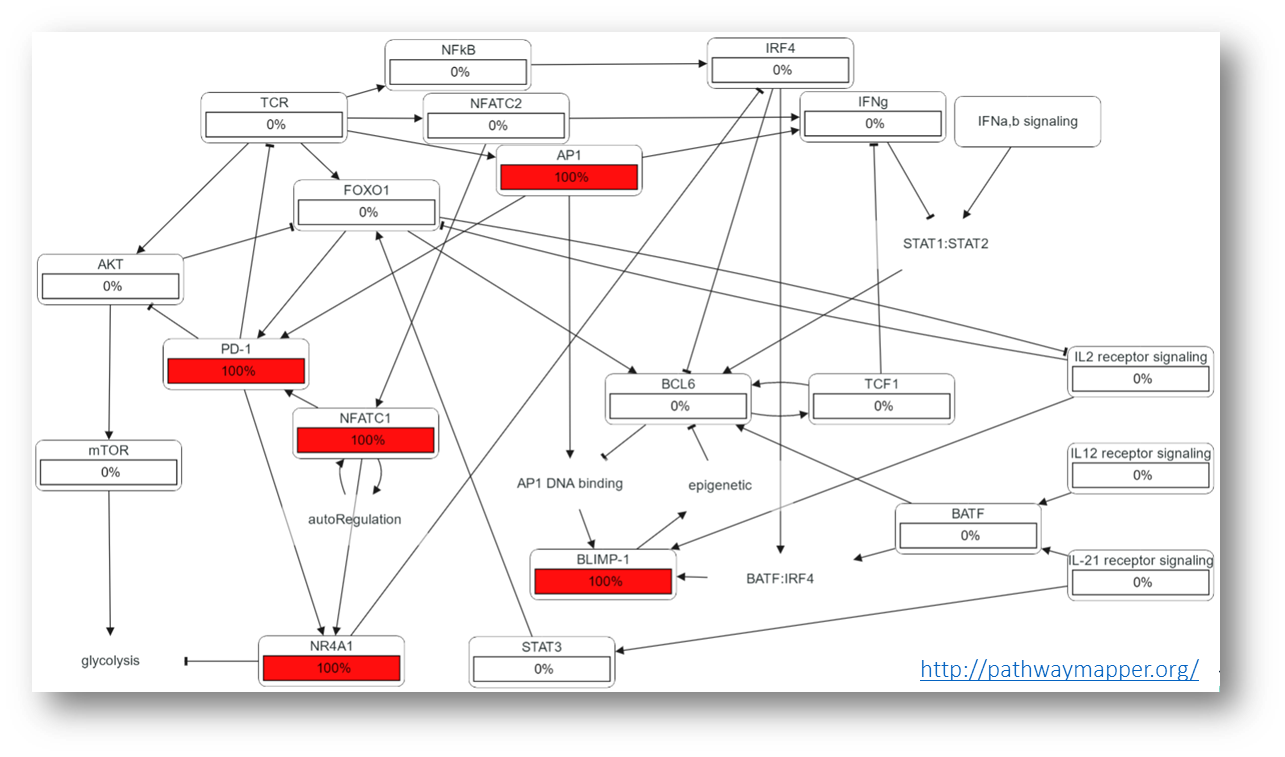


**Supplementary** **Fig. S21**. Four functional network motifs common in T cell exhaustion: positive feedback, negative feedback, and feed-forward loops are defined at left and schematically presented at right. Mutual inhibition (bottom row) is a special case of positive feedback, exemplified in the TCE network by the mutual inhibition between the BCL6/TCF-1 axis and
BLIMP-1. The schematic presented here shows a tube resting on a fulcrum at its middle. If the tube is partially filled with a liquid, it becomes bistable. Any shift of the liquid to one side of the tube will lower the corresponding end of the tube, causing more liquid to flow in the same direction.


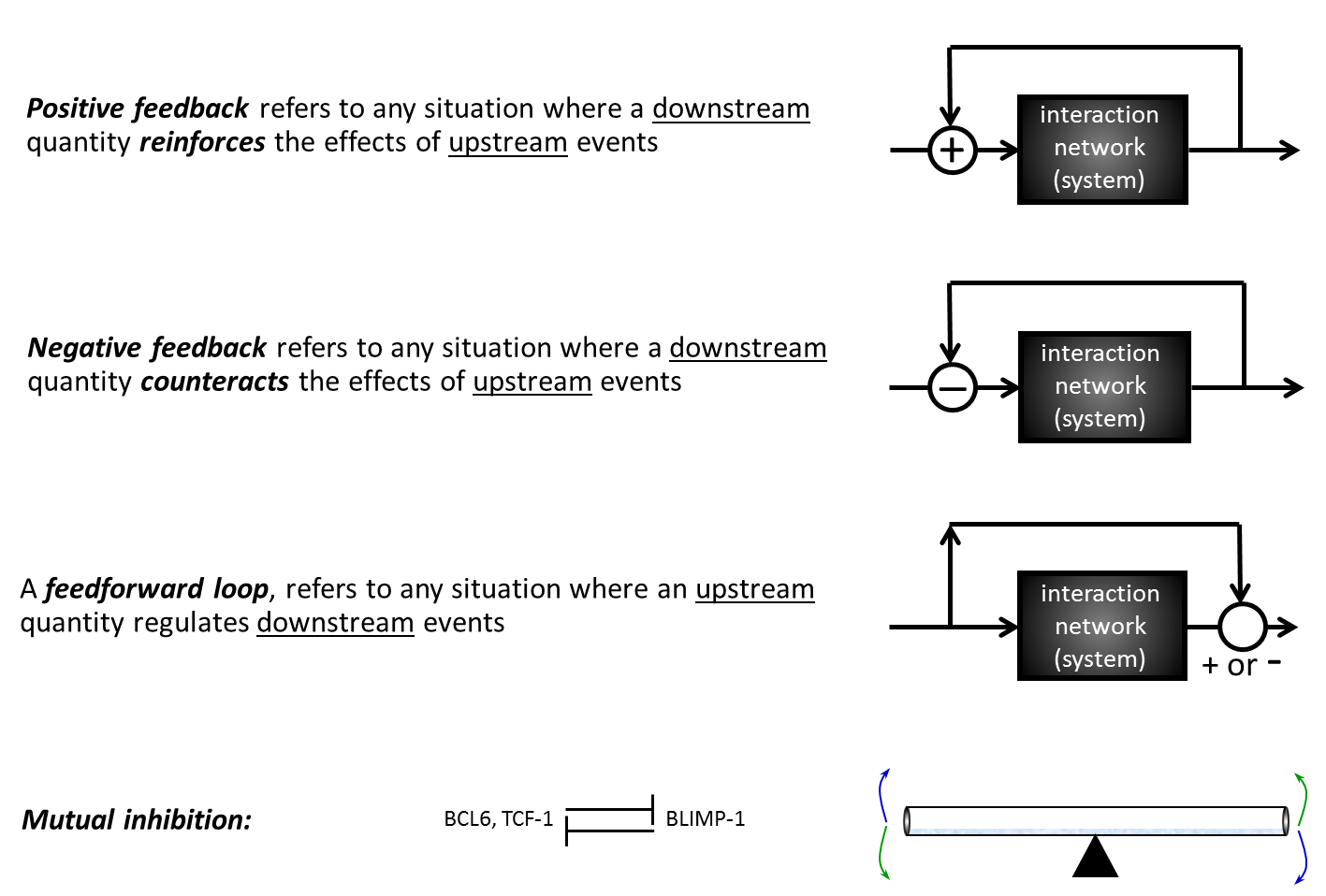


Interaction network (system)

***Positive feedback*** refers to any situation where a downstream quantity ***reinforces*** the effects of upstream events

Interaction network (system)

Interaction network (system)

BLIMP-1

BCL6, TCF-1

***Mutual inhibition:***

A ***feed-forward loop*** refers to any situation where an upstream quantity regulates downstream events

***Negative feedback*** refers to any situation where a downstream quantity ***counteracts*** the effects of upstream events

**Supplementary Fig. S22**. The simplified literature-based network after grouping uninformative isoforms and chains. Note that some gene families/isoforms have been collapsed into single nodes (e.g. NF-κB) unless isoforms are known to play distinct roles (e.g. ID2, ID3) during acute/chronic T cell responses.


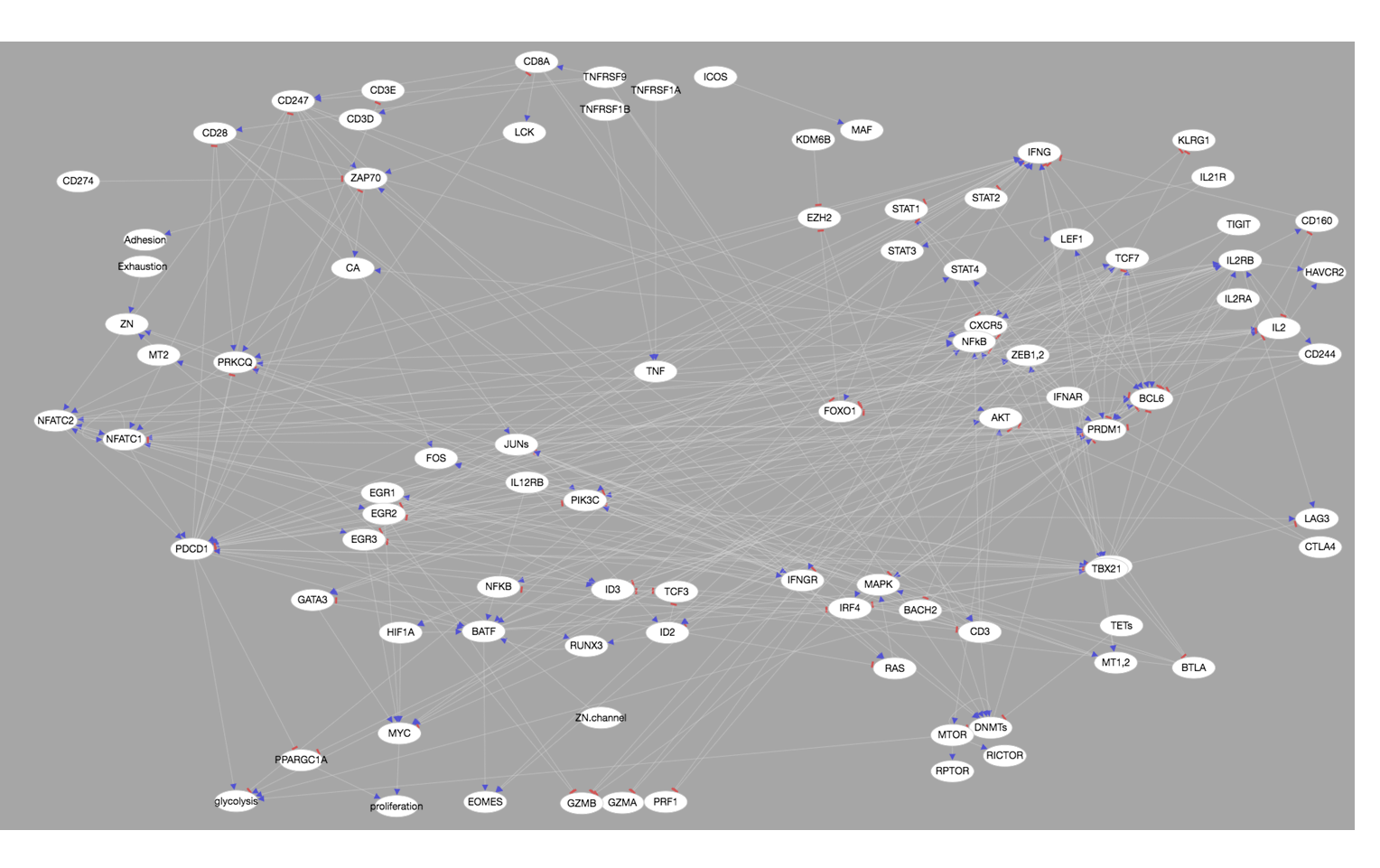


**Supplementary Fig. S23**. **A two-state view of the key gene expression during CD8^+^ T cell activation and exhaustion.** Shown are heatmaps comparing expression profiles of T cell exhaustion (TCE)-associated genes in CD8^+^ T cells from 3 published studies: (**a**) **GSE89307**^7^ data are for CD8^+^ cells in a murine liver cancer model. (**b**) **GSE84105**^8^ and (**c**) **GSE74148**^9^ are from mouse chronic infection models, in which CXCR5^+^ cells were previously identified as being reversibly exhausted, while TIM3^+^ cells were identified as being irreversibly exhausted. For clarity, genes primarily regulated post-transcriptionally in TCE are not shown. The heatmap color scale represents per gene log2 expression Z-scores truncated at +/-1. Each heatmap shows 3 groups of genes, as indicated by the color bar at the left of the heatmaps. Genes marked ‘IR’ (gray) represent immune/inhibitory receptors. Genes marked ‘PP’ (green) have high expression early on following stimulation, and are associated with proliferative and memory-precursor states. Genes marked ‘EE’ (purple) have high expression at later CD8^+^ T cell activation periods, and are associated with effector function and irreversible exhaustion. Note the similarities between Tumor Day 5 and Day 7 CD8^+^ cells, and between these states and CXCR5^+^ cells in chronic infection, as well as similarities between Tumor Day 14 and Day 21 CD8^+^ T cells and TIM3^+^ and committed effector cells in chronic infection.


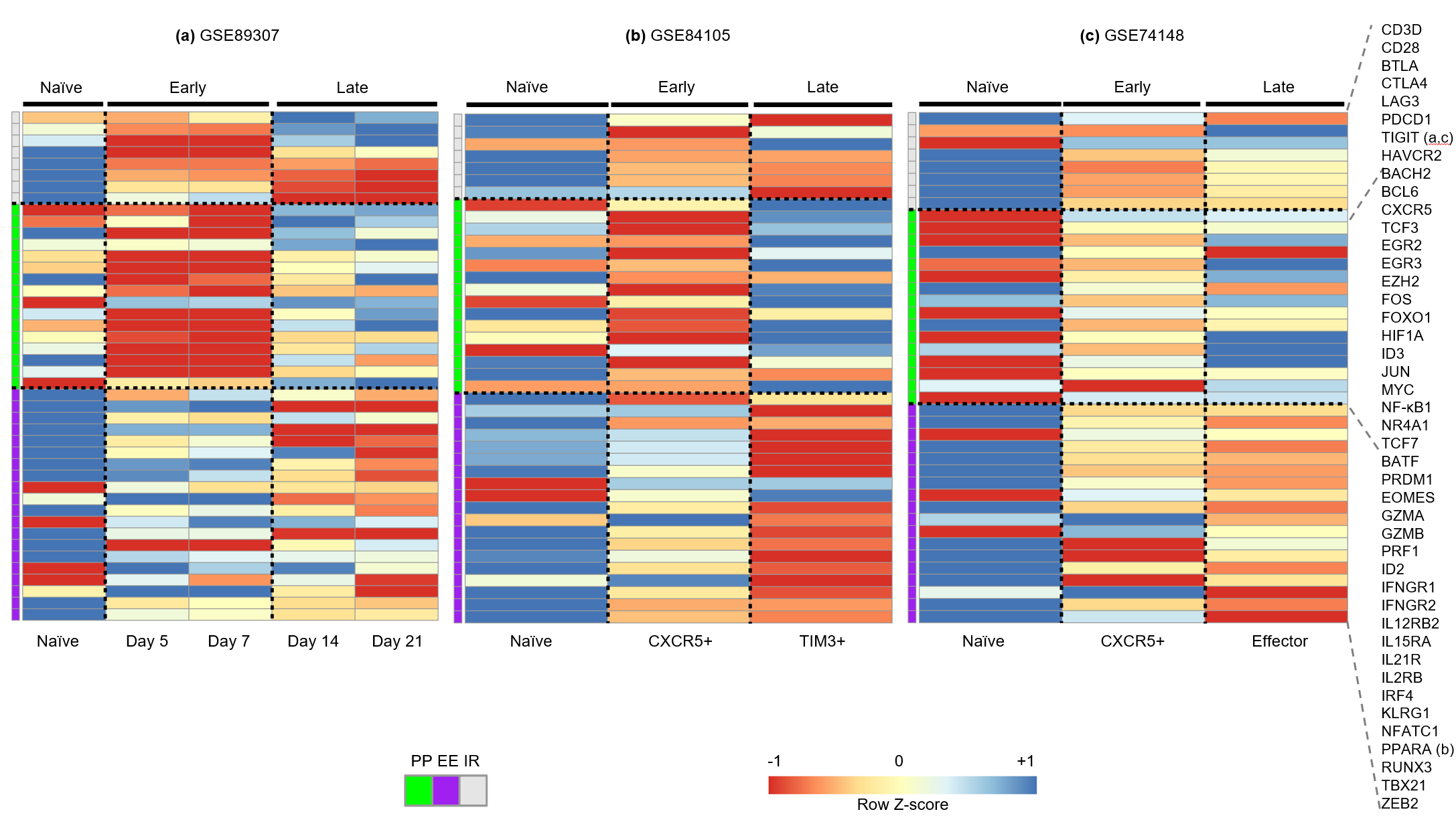


**Supplementary Fig. S24**. **The TCE network can be decomposed and understood as a collection of overlapping functional building blocks.** (**a**) There is widespread mutual inhibition between early (PP) and late (EE) state genes, suggesting the 2 states mutually exclude each other. (**b**) The expression of the PP-state driver gene TCF-1, the onset of restimulation-induced cell death (RICD) via FAS/FASL signaling, and the time of activation of the key EE state genes BLIMP-1 (aka PRDM1), TBET, and ZEB2 are each controlled by multiple, overlapping incoherent feed-forward loop network motifs. Double lines mark slow/delayed processes. The triangular symbol in the FAS/FASL network indicates that IFN-γ signaling is required for upstream factors such as 4-1BB, NFATs, EGRs, and NR4A1 to activate the transcription of FAS and FASL. (**c**) Inhibitory immune receptors implement overlapping negative feedback inhibition of T cell activation. It should be noted that CTLA4, LAG3 and TIGIT additionally exert inhibitory activity via regulatory T cells not shown here to emphasize the negative feedback on CD8/TCR signaling exerted by inhibitory receptors. (**d**) Multiple positive feedback loops reinforce and maintain the late effector/irreversible exhaustion (EE) state.


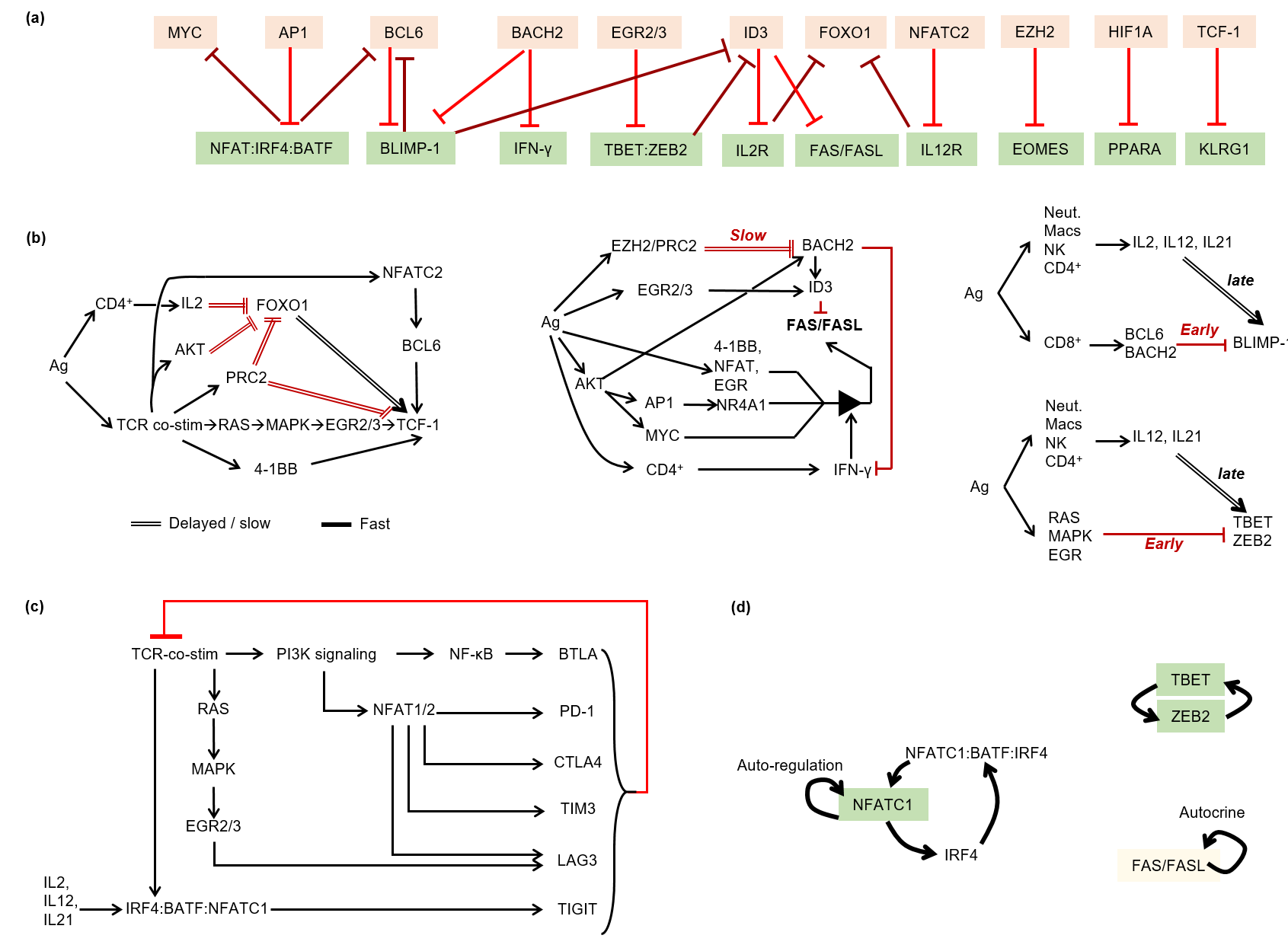


**Supplementary** **Fig. S25**. Single-cell RNA-seq data suggest the mutual repression of *BCL6* and BLIMP-1 (*PRDM1*) is all-or-nothing (bistable). Panel (**a**) shows a theoretical phase portrait for bistable mutual exclusion (adapted from Bolouri^10^). Red arrows show the direction of change in BCL6 and BLIMP-1 for any given pair of values. Example state trajectories are shown in cyan. Note that all trajectories end at one of two possible steady states (at top-left and bottom-right). Inset shows example time courses for BLIMP-1 (blue) and BCL6 (red) activity levels corresponding to the black trajectory in the main figure. (**b**) In 4,482 of 5,063 single CD8^+^ T cells (89%), either BCL6 or BLIMP-1 mRNA is not detected. Inset shows that only 6% of cells have > 4 BCL6 & PRDM1 reads at the same time. Data from Zheng et al^11^.

Log2(PRDM1 + 1)

BCL6 (A.U.)

BLIMP-1 (A.U.)

Log2(BCL6 + 1)

BCL6 < threshold

BLIMP-1
below
detection
threshold

BLIMP-1

BCL6, TCF-1


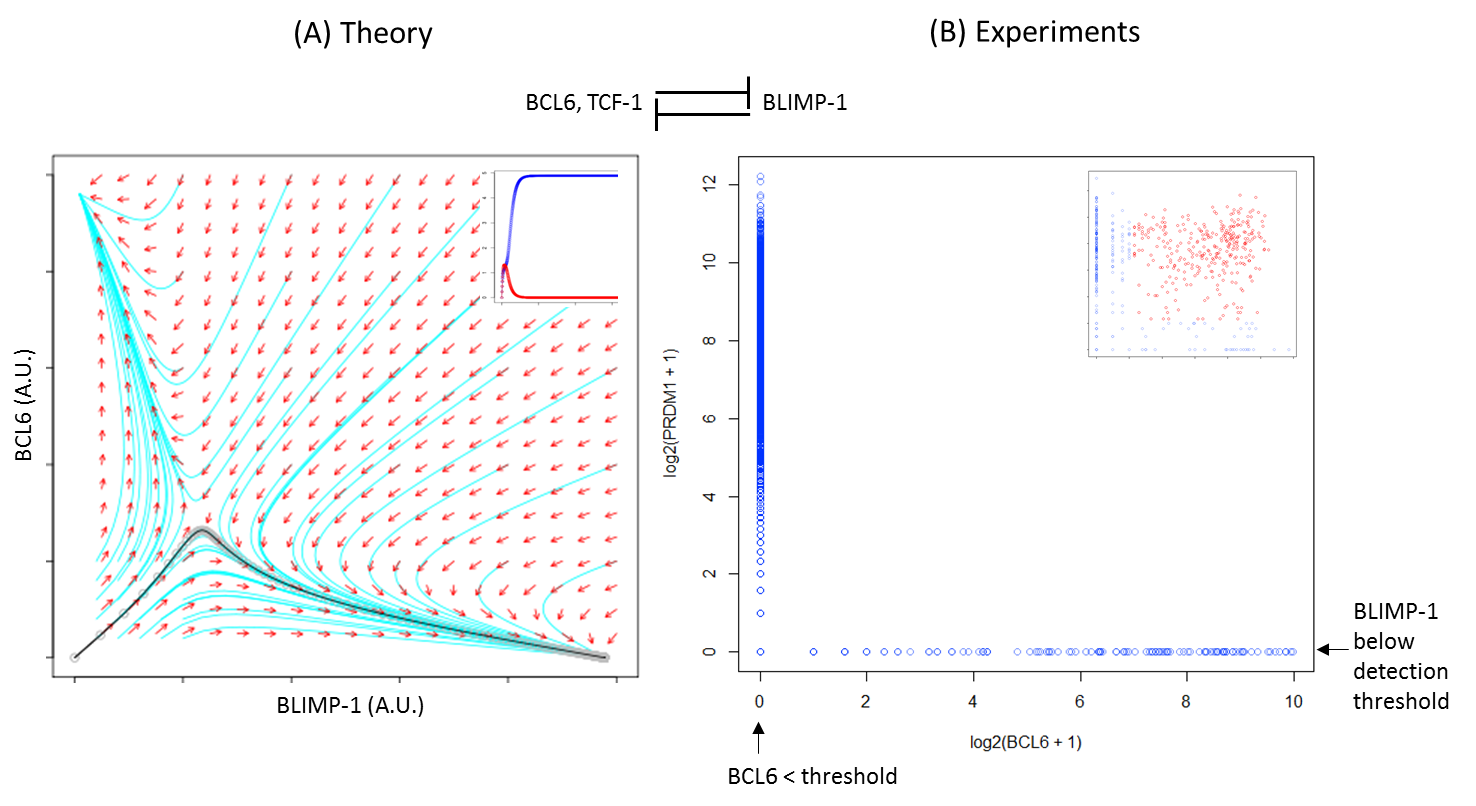


**(a)** Theory

**(b)** Experiments

**Supplementary Fig. S26**. Compared to simple direct regulation (**a**), negative feedback can enable faster responses, and more precise response to stimuli (**b**). ‘*t*_1/2_‘ is the time it takes for a nominal gene (*x*) to reach 50% activity. ‘*b*’ is an activating input. The plots in (**a**, **b**) show how the activity of ‘*x*’ varies with the level of the input ‘*b*’ under direct and negative-feedback regulation. In (**a**), the steady state value of *x* (denoted ‘*x*_ss_’) is a linear function of ‘*b*’. With negative feedback (**b**), *x*_ss_ (the point at which the rate of production of ‘*x*’ - blue and green curves - crosses its loss rate - red line) varies non-linearly with ‘*b*’. As a result, a large change in ‘*b*’ can produce a much smaller change in *x*_ss_. (Rosenfeld et al^12^).


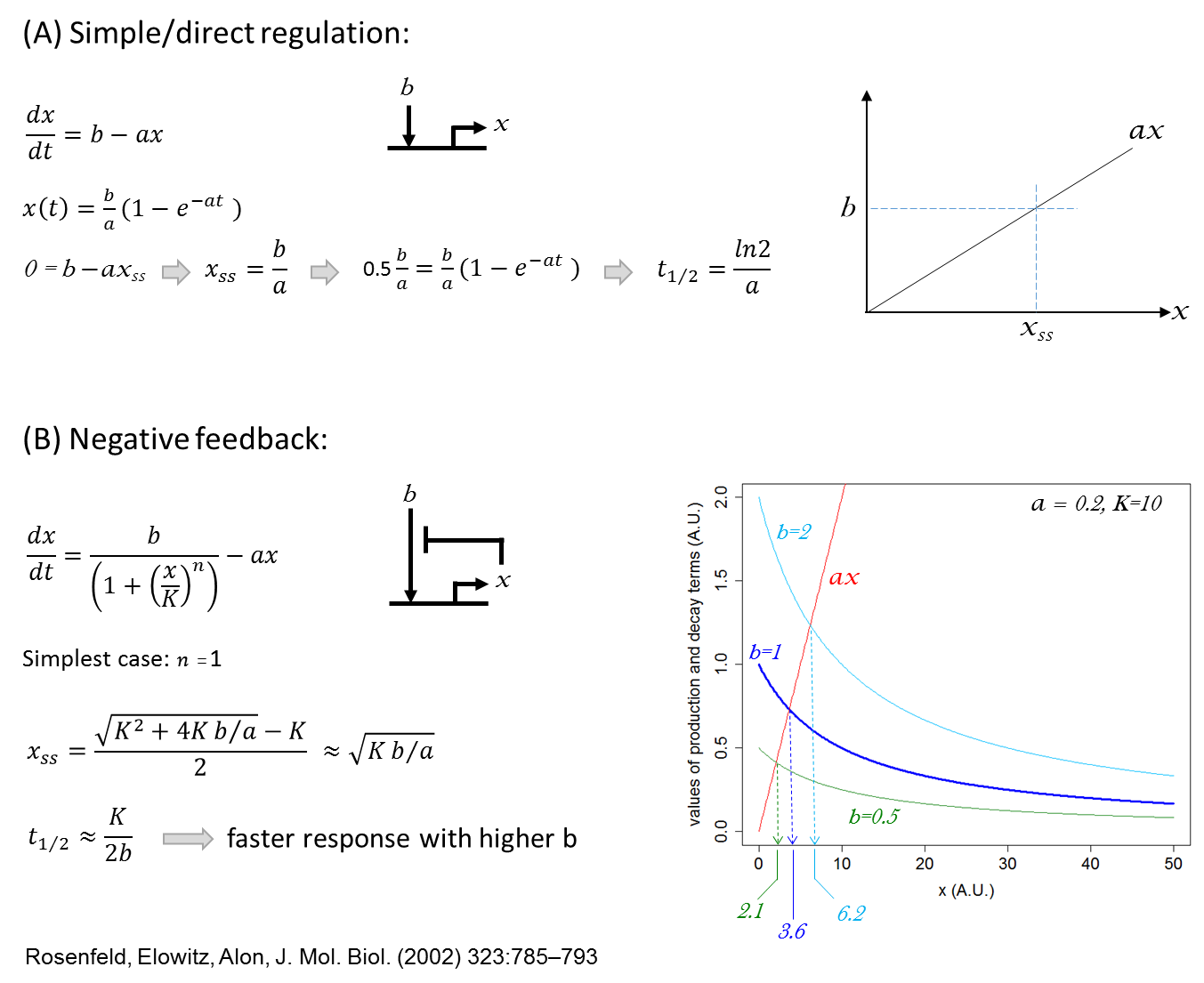


**(a)** Simple/direct regulation:

**(b)** Negative feedback:

Faster response with higher b

Values of production and decay terms (A.U.)

x (A.U.)

Simplest case:

**Supplementary** **Fig. S27**. A simplified simulation model of negative feedback by inhibitory receptors on CD8^+^ T cells demonstrates the potential benefits of the Negative Feedback functional motif. (**a**) and (**b**) show simplified network diagrams. (**c**) Example simulation results demonstrating the speed gain and robustness properties of negative feedback. Model ODE has the form: *y*’ = ((11.175+*x*)/(1+*y*)) - 0.1**y*, where the parameter values are selected arbitrarily for purely illustrative purposes.


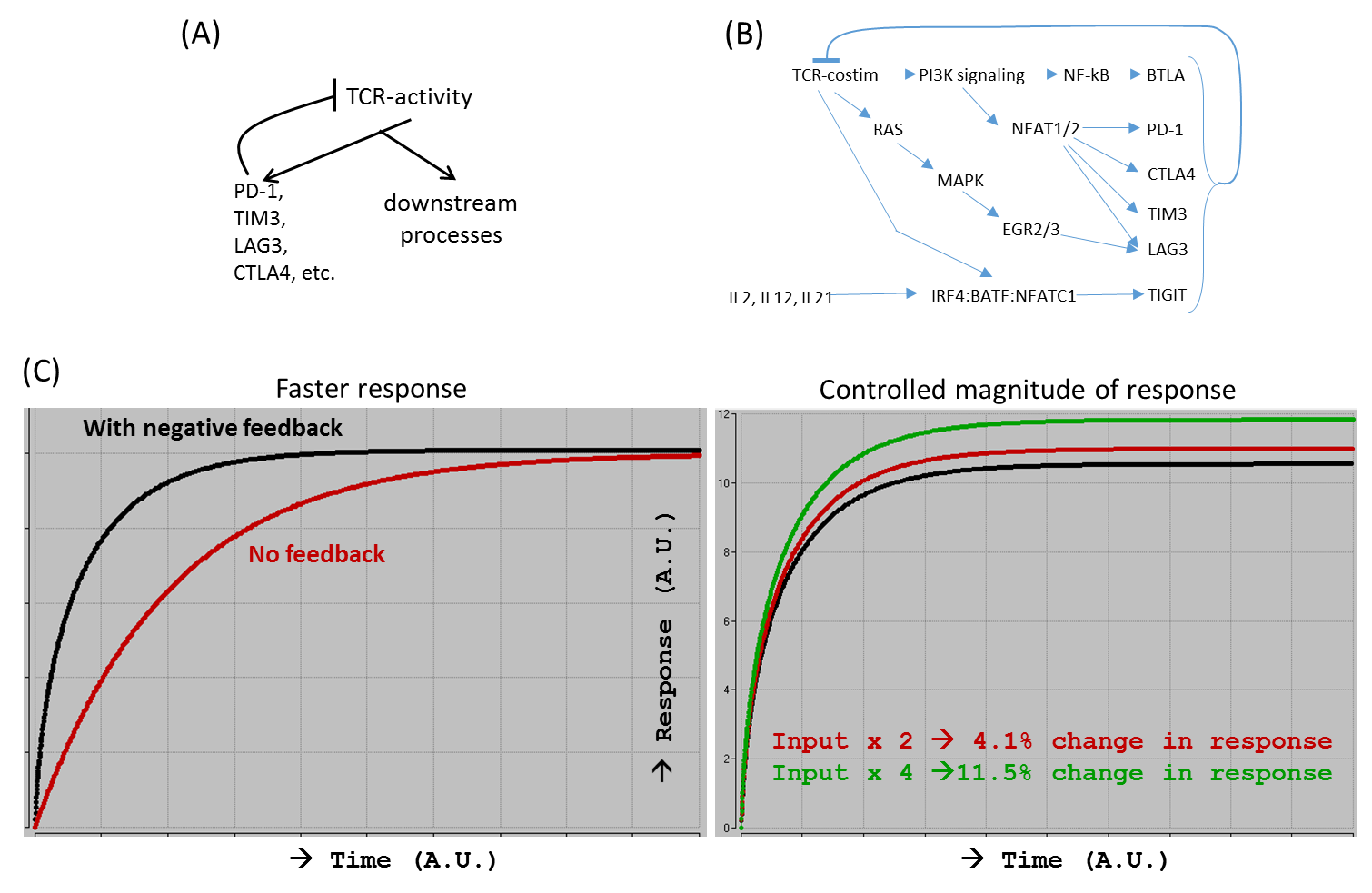


**(a)**

**(b)**

**(c)** Faster response Controlled magnitude of response

Downstream
processes

**Supplementary References (for the Supplementary Figures)**

1. Misstear, K. et al. Suppression of antigen-specific T cell responses by the Kaposi's sarcoma-associated herpesvirus viral OX2 protein and its cellular orthologue, CD200. *J. Virol.* **86,** 6246–6257 (2012).

2. Rosenblum, M. D. et al. CD200 is a novel p53-target gene involved in apoptosis-associated immune tolerance. *Blood* **103,** 2691–2698 (2004).

3. Fernández-Sáiz, V. et al. SCFFbxo9 and CK2 direct the cellular response to growth factor withdrawal via Tel2/Tti1 degradation and promote survival in multiple myeloma. *Nat. Cell. Biol.* **15,** 72–81 (2013).

4. Kurachi, H. et al. Human SPA-1 gene product selectively expressed in lymphoid tissues is a specific GTPase-activating protein for Rap1 and Rap2. Segregate expression profiles from a rap1GAP gene product. *J. Biol. Chem.* **272,** 28081–28088 (1997).

5. Albert, I., Thakar, J., Li, S., Zhang, R. & Albert, R. Boolean network simulations for life scientists. *Source Code Biol. Med*. **3**, 16 (2008).

6. Bahceci, I. et al. PathwayMapper: a collaborative visual web editor for cancer pathways and genomic data. *Bioinformatics*. **33**, 2238–2240 (2017).

7. Philip, M. et al. Chromatin states define tumour-specific T cell dysfunction and reprogramming. *Nature* **545,** 452–456 (2017).

8. Im, S. J. et al. Defining CD8^+^ T cells that provide the proliferative burst after PD-1 therapy. *Nature* **537,** 417–421 (2016).

9. He, R. et al. Follicular CXCR5-expressing CD8^+^ T cells curtail chronic viral infection. *Nature* **537,** 412–428 (2016).

10. Bolouri, H. *Computational Modeling of Gene Regulatory Networks – a Primer* (Imperial College Press, London, 2008).

11. Zheng, C. et al. Landscape of infiltrating T cells in liver cancer revealed by single-cell sequencing. *Cell* **169,** 1342–1356.e16 (2017).

12. Rosenfeld, N., Elowitz, M. B. & Alon, U. Negative autoregulation speeds the response times of transcription networks. *J. Mol. Biol.* **323,** 785–793 (2002).
